# Age‐dependent NMDA receptor function is regulated by the amyloid precursor protein

**DOI:** 10.1111/acel.13778

**Published:** 2023-01-26

**Authors:** Joana Rajão‐Saraiva, Jade Dunot, Aurore Ribera, Mariana Temido‐Ferreira, Joana E. Coelho, Svenja König, Sébastien Moreno, Francisco J. Enguita, Michael Willem, Stefan Kins, Hélène Marie, Luísa V. Lopes, Paula A. Pousinha

**Affiliations:** ^1^ Instituto de Medicina Molecular João Lobo Antunes, Faculdade de Medicina de Lisboa Universidade de Lisboa Lisboa Portugal; ^2^ University Côte d' Azur, Centre National de la Recherche Scientifique (CNRS) UMR 7275, Institut de Pharmacologie Moléculaire et Cellulaire (IPMC) Valbonne France; ^3^ Division of Human Biology and Human Genetics University of Kaiserslautern Kaiserslautern Germany; ^4^ Biomedical Center (BMC), Division of Metabolic Biochemistry, Faculty of Medicine Ludwig‐Maximilians‐Universität München Munich Germany

**Keywords:** aging, AICD, Alzheimer's disease, APP, excitatory synapse, Fe65, GluN2B, hippocampus, NMDA receptor, postnatal development

## Abstract

N‐methyl‐D‐aspartate receptors (NMDARs) are critical for the maturation and plasticity of glutamatergic synapses. In the hippocampus, NMDARs mainly contain GluN2A and/or GluN2B regulatory subunits. The amyloid precursor protein (APP) has emerged as a putative regulator of NMDARs, but the impact of this interaction to their function is largely unknown. By combining patch‐clamp electrophysiology and molecular approaches, we unravel a dual mechanism by which APP controls GluN2B‐NMDARs, depending on the life stage. We show that APP is highly abundant specifically at the postnatal postsynapse. It interacts with GluN2B‐NMDARs, controlling its synaptic content and mediated currents, both in infant mice and primary neuronal cultures. Upon aging, the APP amyloidogenic‐derived C‐terminal fragments, rather than APP full‐length, contribute to aberrant GluN2B‐NMDAR currents. Accordingly, we found that the APP processing is increased upon aging, both in mice and human brain. Interfering with stability or production of the APP intracellular domain normalized the GluN2B‐NMDARs currents. While the first mechanism might be essential for synaptic maturation during development, the latter could contribute to age‐related synaptic impairments.

## Graphical abstract

Rajão‐Saraiva et al. identified the amyloid precursor protein (APP) as an important regulator of NMDA receptors, acting through a dual age‐dependent mechanism. During postnatal development, APP controls GluN2B‐NMDAR synaptic content and currents, potentially contributing to synaptic maturation. Upon aging, the APP amyloidogenic intracellular fragments contribute to aberrant GluN2B‐NMDAR currents. This work highlights the importance of keeping APP processing under tight control, to ensure the normal functioning of glutamatergic synapses, being particularly relevant to understand age‐related synaptic impairments and Alzheimer's disease.

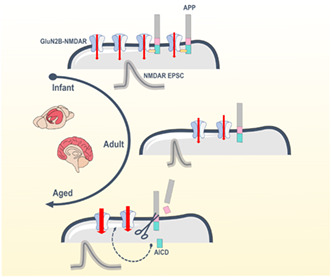

**Abbreviations**
3xTg‐AD: triple transgenic mouse model of Alzheimer's disease
AD: Alzheimer's disease
AICD: APP intracellular domain
APLP1: amyloid precursor‐like protein 1
APLP2: amyloid precursor‐like protein 2
APP: Amyloid precursor protein
APPswe: amyloid precursor protein with Swedish mutation
Aslow: amplitude of the slow component
Aβ: amyloid beta
BACE1: β‐secretase 1
BI: β‐secretase 1 inhibitor
Ca2+: Calcium
CTFs: APP C‐terminal fragments
CTFα: APP C‐terminal fragment α
CTFβ: APP C‐terminal fragment β
cTKO: triple conditional knockout
DIV: days in vitro
EPSCs: excitatory postsynaptic currents
fEPSPs: field excitatory postsynaptic potentials
Ifen: ifenprodil
IPTG: Isopropyl β‐D‐1‐thiogalactopyranoside
LTD: long‐term depression
LTP: long‐term potentiation
MAPT: microtubule‐associated protein tau
NMDAR: N‐methyl‐D‐aspartate receptors
NS1: nonsilencing sequence
pGluN2B: phosphorylated GluN2B
PSD: postsynaptic density
PSD‐95: Postsynaptic density protein 95
PSEN1: presenilin‐1
PTB1‐p: PTB1 peptide
PTB2‐p: PTB2 peptide
PVDF: polyvinylidene fluoride
ROI: region of interest
RT: room temperature
sAPPα: secreted APP α
sAPPβ: secreted APP β
SEM : standard error of the mean
shAPP: short‐hairpin RNA against APP
shCTR: control short‐hairpin RNA
shRNA: short‐hairpin RNA
τweighted: weighted component

## 1 INTRODUCTION

The activation of N‐methyl‐D‐aspartate receptors (NMDARs) at glutamatergic synapses results in calcium influx into neurons, activating downstream signaling pathways (Hardingham et al., 1999). Due to their voltage‐dependent block by magnesium, NMDARs act as coincidence detectors for pre‐ and postsynaptic activity since receptor activation requires glutamate release and strong membrane depolarization (Nowak et al., 1984). This process is critical for the formation, maturation, maintenance, and plasticity of glutamatergic synapses, thereby contributing to development, learning, and memory processes (Gambrill & Barria, 2011; Morris et al., 1986). At the same time, NMDAR dysfunction is associated with several pathological conditions, including neurodevelopmental disorders (Burnashev & Szepetowski, 2015) and aged‐related neurodegenerative diseases such as Alzheimer's disease (AD; Zhou & Sheng, 2013).

One of the main factors that determine NMDAR properties is their subunit composition. N‐methyl‐D‐aspartate receptors are heterotetramers composed of two obligatory GluN1 subunits and two GluN2 (A–D) or GluN3 (A and B) subunits (Paoletti et al., 2013). In the hippocampus and cortex, NMDARs mainly contain GluN2A and/or GluN2B regulatory subunits (Monyer et al., 1994). GluN2B‐NMDARs differ from the GluN2A subtype due to their slower deactivation kinetics, higher Ca^2+^ charge per unit of current, and specific intracellular interactors (Paoletti et al., 2013). Therefore, the synaptic GluN2B/GluN2A ratio determines the consequences of NMDAR activation, including total calcium influx and downstream signaling. This ratio is not static, changing not only in response to neuronal activity and sensory experience during postnatal development, but also in adult synapses (Paoletti et al., 2013).

During development, glutamatergic synaptic transmission in nascent synapses is mainly mediated by GluN2B‐NMDARs (Bellone & Nicoll, 2007). When there is strong or synchronous neuronal activity, resulting in enough calcium entry through GluN2B‐NMDARs (Adesnik et al., 2008), synapses undergo maturation, leading to alterations in the composition of postsynaptic receptors, namely a shift from a predominance of GluN2B to GluN2A‐NMDARs (Williams et al., 1993; Bar‐Shira et al., 2015). Upon adulthood, both NMDAR subtypes contribute for normal calcium signaling (Sobczyk et al., 2005), synaptic plasticity (Massey et al., 2004), and memory formation (von Engelhardt et al., 2008; Bannerman et al., 2008). However, these NMDAR‐dependent processes become dysregulated upon aging, resulting in elevated postsynaptic calcium levels (Thibault et al., 2001), alterations in long‐term potentiation/depression (LTP/LTD; Burke & Barnes, 2006; Temido‐Ferreira et al., 2020) and memory deficits (Tombaugh et al., 2002). Based on this, NMDAR gain of function has emerged as a possible explanation for age‐related synaptic impairments (Kumar, 2015). Accordingly, GluN2B present higher retention at aged synapses (al Abed et al., 2020; Zamzow et al., 2013) and an inverse correlation with memory performance (Zhao et al., 2009). Moreover, GluN2B‐NMDAR overactivation and consequent excessive calcium influx are known to contribute to age‐associated neurodegenerative disorders (Ferreira et al., 2017; Hanson et al., 2015). This hypothesis is counterintuitive, as the GluN2B subunit is thought to be the most affected by aging (Magnusson, 2012), showing a decline in expression levels (Magnusson, 2012; Zhao et al., 2009). Further knowledge by electrophysiological examination of functional changes in GluN2B‐NMDARs during the aging process may be the key to understand aging cognitive disabilities.

The amyloid precursor protein (APP) has emerged as a putative NMDAR regulator, since it interacts with NMDARs in rodent brain lysates and in primary neuronal cultures (Cousins et al., 2009; Hoe et al., 2009). Also, we previously showed that in utero silencing of APP causes the loss of GluN2B synaptic contribution in infant mice (Pousinha et al., 2017). Studying the role of APP is not only challenging considering the functional redundancy with members of the same protein family, but also given the multiple APP fragments, generated by secretase cleavage, with specific cellular functions (Müller et al., 2017). The APP family is composed of three highly conserved transmembrane glycoproteins, the APP and amyloid precursor‐like proteins 1 and 2 (APLP1 and APLP2), with overlapping functions (Müller & Zheng, 2012). Amyloid precursor protein undergoes extracellular cleavage mainly by α‐ or β‐secretase (nonamyloidogenic or amyloidogenic pathway, respectively), resulting in the formation of large N‐terminal extracellular fragments of secreted APP (sAPPα or sAPPβ, respectively) and smaller membrane‐bound C‐terminal fragments (Müller et al., 2017). Subsequently, the C‐terminal fragments are subjected to an intramembranous scission by the γ‐secretase complex (Wolfe et al., 1999) to generate the APP intracellular domain (AICD), which can be stabilized through its interaction to the PTB2 domain of Fe65 (Borg et al., 1996; Kimberly et al., 2001). The same cleavage step simultaneously releases p3 (after α‐secretase cleavage) or the AD‐related amyloid‐β peptide (Aβ, after β‐secretase cleavage). Therefore, APP has been mostly studied in the context of AD, while far less is known about its physiological role throughout life. Amyloid precursor protein levels reach their peak during brain development (Kirazov et al., 2001), when it has been suggested to participate in synaptogenesis and clustering of synaptic proteins, as revealed by in vitro overexpression/knockdown studies (Baumkötter et al., 2012). Later in life, a role for APP in synaptic plasticity has been proposed, considering the impairments in LTP and cognitive behavioral performance observed in aged APP‐knockout animals (>10 months), but not in earlier stages (2–4 months; Dawson et al., 1999).

We now explored the mechanism by which APP regulates NMDAR synaptic transmission at different life stages. Combining electrophysiological outputs and molecular approaches, we found a dual mechanism by which APP controls GluN2B‐NMDARs. We identified the APP full‐length protein as a new regulator of glutamatergic transmission in immature synapses, by controlling GluN2B synaptic content and mediated currents during development. In addition, we gathered strong evidence showing that AICD generated by the amyloidogenic pathway modifies NMDAR transmission, favoring the GluN2B synaptic contribution at later life stages. Our work highlights the importance of keeping APP processing under tight control, to ensure the normal functioning of glutamatergic synapses, being particularly relevant to understand age‐related synaptic impairments and AD.

## 2 RESULTS

### 2.1 GluN2B‐NMDAR synaptic contribution is increased in infant and aged mice

**FIGURE 1:**
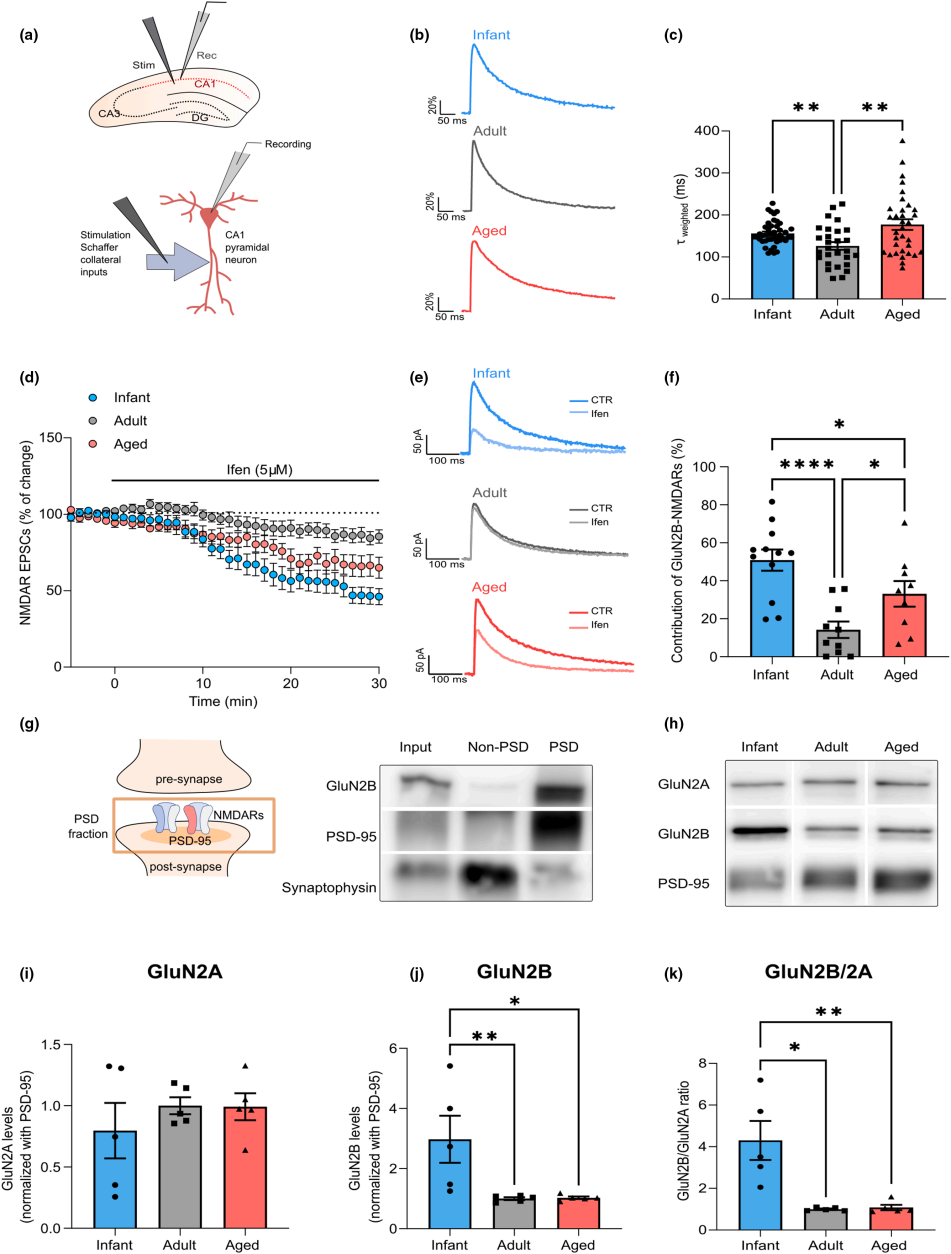
GluN2B‐N‐methyl‐D‐aspartate receptor (NMDAR) synaptic contribution is increased in infant and aged mice. (a) Schematic diagram showing the locations of stimulating and recording electrodes in the hippocampus and in a CA1 pyramidal neuron for whole‐cell patch‐clamp experiments. (b) Comparison of representative whole‐cell patch‐clamp recordings of pharmacologically isolated NMDAR EPSCs, normalized to the peak amplitude (in %), from infant (7–10 days), adult (10–16 weeks), and aged (18–20 months) C57BL/6 wild‐type mice, illustrating differences in deactivation kinetics. (c) The weighted time constant (τweighted) was calculated using the relative contribution of both slow/fast components of NMDAR EPSCs and reflects the overall deactivation kinetics. Results are expressed as the mean ± SEM (Kruskal–Wallis, *p* = 0.004, Kruskal–Wallis statistic = 11.02, followed by uncorrected Dunn's multiple comparisons test, **p < 0.01, *n* = 28–44). (d) Time course of ifenprodil (5 μM) effect on pharmacologically isolated NMDAR EPSC amplitude in CA1 pyramidal neurons, measured by whole‐cell patch clamp in infant, adult, and aged C57BL/6 wild‐type mice. Results are expressed as the mean ± SEM (*n* = 9–12). (e) Traces show NMDAR EPSCs recorded before (CTR) and after 30 min of Ifenprodil 5 μM perfusion (Ifen). (f) GluN2B contribution was calculated as the percentage of change in NMDAR EPSC amplitude after 30 min of ifenprodil perfusion. Results are expressed as the mean ± SEM (One‐way ANOVA, *p* = 0.0002, *F*(2, 28) = 11.37, followed by uncorrected Fisher's LSD's multiple comparisons test, *p < 0.05, ****p < 0.0001, *n* = 9–12). (g) Schematic representation and immunoblotting analysis of postsynaptic density (PSD)‐enriched fractions from the hippocampal tissue of adult C57BL/6 wild‐type mice. Membranes were immunoblotted with antibodies for GluN2B, PSD‐95, and synaptophysin. PSD fractions are enriched in PSD‐95, whereas non‐PSD fractions contain high levels of synaptophysin. (h) Representative Western blot of hippocampal lysates subjected to biochemical fractionation to obtain PSD‐enriched fractions from infant, adult, and aged C57BL/6 mice. Membranes were immunoblotted with antibodies for GluN2A, GluN2B, and PSD‐95. (i, j) Results from PSD‐enriched fractions blots as shown in h) were normalized with PSD‐95 and are expressed as the mean ± SEM relative to the adult group. (i) One‐way ANOVA, *p* = 0.5735, *F*(2, 12) = 0.5825, *n* = 5). (j) One‐way ANOVA (*p* = 0.014, *F*(2, 12) = 6.224), followed by Uncorrected Fisher's LSD's multiple comparisons test, *p < 0.05, **p < 0.01, *n* = 5). (k) Results show the GluN2B/GluN2A in PSD‐enriched fractions blots as shown in h) and are expressed as the mean ± SEM relative to the adult group (Kruskal–Wallis, *p* = 0.0024, Kruskal–Wallis statistic = 9.42, followed by Uncorrected Dunn's multiple comparisons test, *p < 0.05, **p < 0.01, *n* = 5). The full statistical analysis and Western Blot membranes are provided in the Supporting Information.

We recorded pharmacologically isolated NMDAR excitatory postsynaptic currents (EPSCs) in CA1 pyramidal cells in the hippocampus, evoked by electrical stimulation of Schaffer collaterals (Figure 1a) in C57BL/6 mice at different ages: infant (7–10 days), adult (10–16 weeks), and aged (18–20 months). To investigate whether age‐mediated alterations of NMDAR currents were due to modifications of NMDAR subunit composition, we measured the time constant for the weighted component (τ_weighted_) of NMDAR EPSC deactivation kinetics. We found that NMDAR EPSC deactivation kinetics are age‐dependent, since the τ_weighted_ was higher in infant (156.2 ms ± 4.20) and aged mice (177.0 ms ± 12.66), compared with the reference group of adults (126.3 ms ± 9.11; Figure 1b,c). We also found an increase in the relative amplitude of the slow component (A_slow_) for infant and aged mice (Figure S1a).

Since GluN1/GluN2B heterodimers display slower deactivation kinetics than GluN1/GluN2A heteromers (Paoletti et al., 2013), this suggests that the GluN2B contribution to NMDAR EPSCs is higher at infant and aged life stages, when compared to adults. To test this hypothesis, we evaluated the effect of the selective GluN2B inhibitor, ifenprodil (5 μM), on NMDAR EPSCs. We found that the contribution of GluN2B to NMDAR EPSCs is of 50.88% ± 5.58 in infants, decreased to 14.20% ± 4.32 in adults, and increased to 33.11% ± 6.72 in aged mice, as depicted in Figure 1d–f.

We then correlated these effects with the GluN2B/GluN2A ratio measured in the postsynaptic density (PSD‐enriched fractions; Figure 1g). The synaptic ratio of GluN2B/GluN2A was approximately four times higher in infant mice, than in adult and aged mice (Figure 1k), correlating with the increase in GluN2B synaptic contribution. A similar pattern was observed both in whole lysates (Figure 1f–i) and in PSD‐95 immunoprecipitates (Figure 1j–m). The increase in GluN2B/A ratio in infants possibly reflects the lower expression levels of GluN2A at this age, while GluN2B synaptic levels reach their peak at this stage (Figure 1h–j; Figure S1b,c,f–m).

We also found that the levels of GluN2B phosphorylation (pGluN2B) at the Y1472 residue, known to enhance GluN2B‐PSD95 binding (Rong et al., 2001), are reduced in infant mice and more stable in adult/aged stages (Figure 1d,e). When analyzing human postmortem tissue at different ages (21–89 years old), we found a tendency for an inverse correlation of total GluN2B levels with aging (Figure 1n,o).

These data demonstrate that NMDARs contribute to synaptic transmission in an age‐dependent manner, whereby GluN2B contribution is higher in both infant and aged synapses.

### 2.2 APP interacts and regulates GluN2B‐NMDARs at immature synapses

There are several mechanisms and protein interactors able to regulate NMDAR synaptic transmission and GluN2B/A contribution. The APP has emerged as a potential candidate, following reports by our group and others that APP interacts, and regulates NMDAR surface levels and currents (Cousins et al., 2009; Hoe et al., 2009; Pousinha et al., 2017).

**FIGURE 2:**
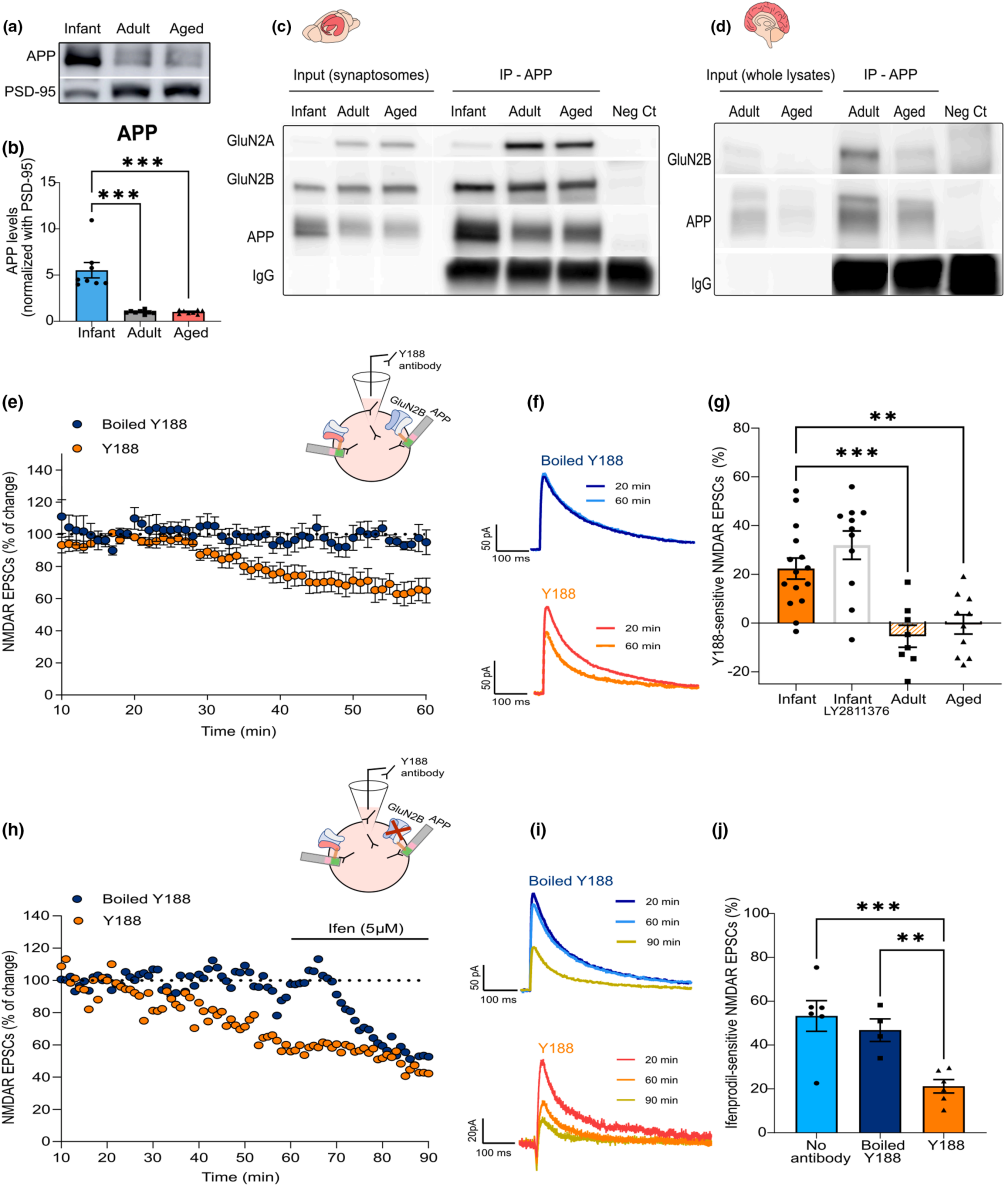
Amyloid precursor protein (APP) interacts and regulates GluN2B‐N‐methyl‐D‐aspartate receptors (NMDARs) at immature synapses. (a) Representative Western blot of hippocampal postsynaptic density (PSD)‐enriched fractions from infant (7–10 days), adult (10–16 weeks), and aged (18–20 months) wild‐type C57BL/6 mice. Membranes were immunoblotted with antibodies for APP and PSD‐95. (b) Results from PSD‐enriched fractions blots as shown in a) show APP levels normalized with PSD‐95 and are expressed as the mean ± SEM relative to the adult group (Kruskal–Wallis, *p* = 0.0005, Kruskal–Wallis statistic = 15.37, followed by uncorrected Dunn's test, ***p < 0.001, *n* = 8). (c) Representative Western blot of synaptosome fractions from the hippocampi of infant, adult, and aged wild‐type C57BL/6 mice immunoprecipitated for APP. Membranes were immunoblotted with antibodies for GluN2A, GluN2B, and APP. (d) Representative Western blot of postmortem brain tissue (prefrontal cortex) from human subjects (adult = 22 and aged = 89 years old) immunoprecipitated for APP. Membranes were immunoblotted with antibodies for GluN2B and APP. (e) Time course of NMDAR EPSC amplitude measured by whole‐cell patch clamp in CA1 pyramidal neurons of infant C57BL/6 wild‐type mice during 60 min of incubation with an antibody against the APP C‐terminal (Y188). In the control condition, the antibody was heat‐inactivated (boiled Y188). Results are expressed as the mean ± SEM (*n* = 6–10). The schematic diagram shows the strategy used to mask the APP C‐terminal domain—the antibody was added to the intracellular solution in the patch pipette to diffuse into the intracellular space. (f) Traces show NMDAR EPSCs recorded at 20 min (baseline) and 60 min. The Y188 antibody was inside the patch pipette during the whole course of the experiment (60 min). (g) The percentage of Y188‐sensitive NMDAR EPSCs was determined for infant mice with or without treatment with a BACE1 inhibitor (LY2811376, administration 12 h prior to patch‐clamp recordings), adult, and aged C57BL/6 wild‐type mice. The effect was calculated comparing the baseline amplitude (20 min) with the final amplitude (60 min) and normalized with the control condition (boiled Y188) for each age. Results are expressed as the mean ± SEM (One‐way ANOVA, *p* < 0.0001, *F*(3, 40) = 12.65, followed by uncorrected Fisher's LSD's multiple comparisons test, **p < 0.01, ***p < 0.001, *n* = 8–15). (h) Time course of NMDAR EPSC amplitude measured by whole‐cell patch clamp in CA1 pyramidal neurons of infant C57BL/6 wild‐type mice during 90 min of incubation with the Y188 antibody and perfusion with ifenprodil (5 μM) at 60–90 min. In the control condition, the antibody was heat‐inactivated (boiled Y188; *n* = 1). The schematic diagram shows the strategy used to block APP (Y188 antibody in the patch pipette) and to inhibit GluN2B‐NMDAR (ifenprodil perfusion). (i) Traces show NMDAR EPSCs recorded at 20 min (baseline), 60 min, and 90 min. The Y188 antibody was inside the patch pipette during the whole course of the experiment (90 min), whereas Ifenprodil perfusion occurred from 60 to 90 min. (j) The percentage of ifenprodil‐sensitive NMDAR EPSCs in infant mice was calculated comparing the amplitude at 60 min with the final amplitude (90 min). The effect of ifenprodil on NMDAR EPSCs was calculated in neurons without antibody incubation (No antibody, used as reference), incubated with the Y188 antibody (Y188) or the heat‐inactivated antibody (boiled Y188) for 90 min. Results are expressed as the mean ± SEM (one‐way ANOVA, *p* = 0.0019, *F*(2, 13) = 10.59, followed by uncorrected Fisher's LSD multiple comparisons test, ***p < 0.001, **p < 0.01, *n* = 4–6). The full statistical analysis and Western Blot membranes are provided in the Supporting Information.

We detected APP in PSD‐enriched fractions at all ages and observed a fivefold increase in infants compared with adults, whereas the APP postsynaptic levels remain low in aged synapses (Figure 2a,b; Figure S2a).

In addition, NMDARs co‐immunoprecipitated with APP in whole lysates (Figure S2b), and isolated synaptosomes (Figure 2c) from infant, adult, and aged mice. This interaction is established with GluN2B over GluN2A during postnatal development, and occurs with both GluN2A and GluN2B in adult and aged animals (Figure 2c; Figure S2b–f). More importantly, this interaction was detected in human postmortem brain tissue (Figure 2d).

We previously reported that the loss of GluN2B synaptic contribution following in utero silencing of APP could be reverted by delivering the AICD peptide to the neurons (Pousinha et al., 2017), thus suggesting that APP—NMDR interaction occurs through AICD. We therefore interfered with APP—NMDAR interaction while recording NMDAR EPSCs, by introducing an APP C‐terminal antibody (clone Y188, 2.5 nM) in the recording pipette (Figure 2e). We observed a reduction of 21.11 ± 4.47% in NMDAR EPSCs in infant mice, compared with the control condition (boiled Y188 antibody; as illustrated in Figure 2e–g), not amplified by increasing the Y188 antibody concentration to 5 nM (Figure S2g). This effect was not modified by preventing the APP processing through the oral administration of a β‐secretase 1 (BACE 1; BI) inhibitor 12 h prior to patch‐clamp recordings (LY2811376; 100 mg/kg; Figure 2g; Figure S2h).

To test whether this response is mediated by GluN2B‐NMDARs, we perfused hippocampal slices with a GluN2B selective antagonist, ifenprodil (5 μM), for 30 min at the end of the experiment (60–90 min; Figure 2h). As shown, the effect of ifenprodil was reduced in neurons previously incubated with the Y188 antibody, when comparing to the control condition (19.76 ± 3.29 vs. 46.82 ± 5.16%; Figure 2h–j). BY contrast, we found no alterations in NMDAR‐mediated currents in adult and aged mice by blocking APP (Figure 2g).

In conclusion, these results show that APP interacts with GluN2B at immature synapses, thus regulating their contribution to NMDAR EPSCs.

### 2.3 APP modulates the GluN2B‐NMDAR synaptic content in immature synapses

**FIGURE 3:**
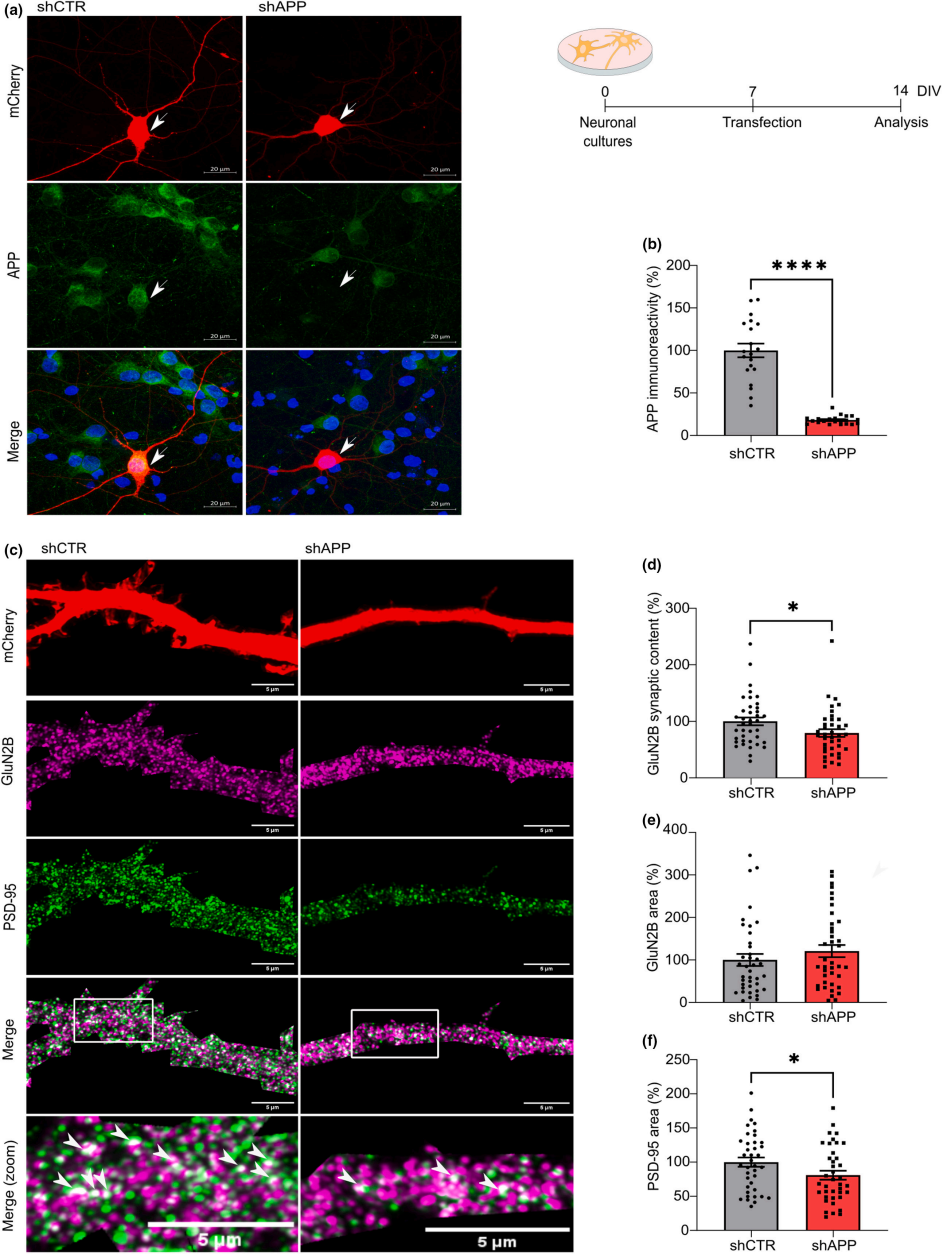
Amyloid precursor protein (APP) modulates the GluN2B‐N‐methyl‐D‐aspartate receptor (NMDAR) synaptic content in immature neurons. (a) Representative immunocytochemistry analysis of APP immunofluorescence in rat primary neuronal cultures (14 days in vitro (DIV)) transfected with shAPP or the respective control (shCTR) at DIV7, as indicated in the timeline. mCherry (reporter plasmid) is shown in red, APP is labeled in green and cell nuclei are stained with Hoechst in blue. Transfected neurons are indicated by arrows. Scale bars represent 20 μm. (b) APP immunoreactivity (%) in transfected neurons is expressed as the mean ± SEM, using the control condition as reference (Mann–Whitney test, ****p < 0.0001, *n* = 20–21 cells, three independent cultures). (c) Representative immunocytochemistry analysis of rat primary neuronal cultures (DIV14) transfected with shAPP or the respective control (shCTR) at DIV7. mCherry (reporter plasmid), labeled in red, was used to identify dendrites of transfected neurons. GluN2B is shown in magenta and postsynaptic density (PSD)‐95 is labeled in green. Higher magnification images are shown at the bottom, with arrows indicating GluN2B/PSD95 co‐localization. Scale bars represent 5 μm. (d and e) Results show GluN2B synaptic content and GluN2B total area in dendrites of transfected neurons. The synaptic content was calculated as the area of GluN2B‐PSD co‐localization normalized with the total area of GluN2B. Results are expressed as the mean ± SEM, using the control condition as reference (%; Mann–Whitney test, *p < 0.05, *n* = 39, three independent cultures). (f) Results show PSD‐95 total area in dendrites of transfected neurons. Results are expressed as the mean ± SEM, using the control condition as reference (%; Unpaired *t* test, *p < 0.05, *n* = 39, 3 independent cultures). The full statistical analysis is provided in the Supporting Information.

The reported impact of APP on NMDAR transmission suggested a role for APP in regulating NMDARs during postnatal development. To assess the outcomes of APP depletion in immature neurons, we transfected primary neuronal cultures (7 days in vitro (DIV)) with a plasmid encoding a short‐hairpin RNA (shRNA) sequence against APP (shAPP) or the respective control (shRNA with no silencing effect, shCTR). The APP knockdown was efficient, leading to a reduction of approximately 80% in APP immunoreactivity, when comparing to the control condition, evaluated 7 days post‐transfection (14 DIV; Figure 3a,b).

Amyloid precursor protein‐depleted neurons showed a significant reduction in the percentage of GluN2B clusters that co‐localize with PSD‐95 (postsynaptic marker; approximately 20%; Figure 3c,d). The GluN2B relative dendritic area, average particle size, and fluorescent density were not altered by APP depletion, indicating that GluN2B total levels/area and clustering remained constant (Figure 3c,e; Figure S3a,b).

Considering the impact of GluN2B on synaptic maturation, in which PSD‐95 recruitment is a crucial step (Elias et al., 2008), we also evaluated whether this process is impaired in APP‐depleted neurons. We found a reduction in PSD‐95 dendritic area and average particle size (Figure 3c,f; Figure S3c), whereas the fluorescence density was not altered (Figure S3d).

These findings show that APP regulates GluN2B synaptic content and PSD‐95 clustering in immature neurons.

### 2.4 Age‐related increase in βAPP processing contributes to higher GluN2B synaptic contribution

Since the increase in GluN2B contribution to NMDAR EPSCs in aged mice is not correlated with APP postsynaptic levels or APP‐NMDAR interaction, we hypothesized that APP in its full‐length form is not responsible for these alterations. In fact, we had previously reported that an APP‐derived fragment, the AICD, has the ability to regulate synaptic GluN2B in CA1 pyramidal neurons (Pousinha et al., 2017).

**FIGURE 4:**
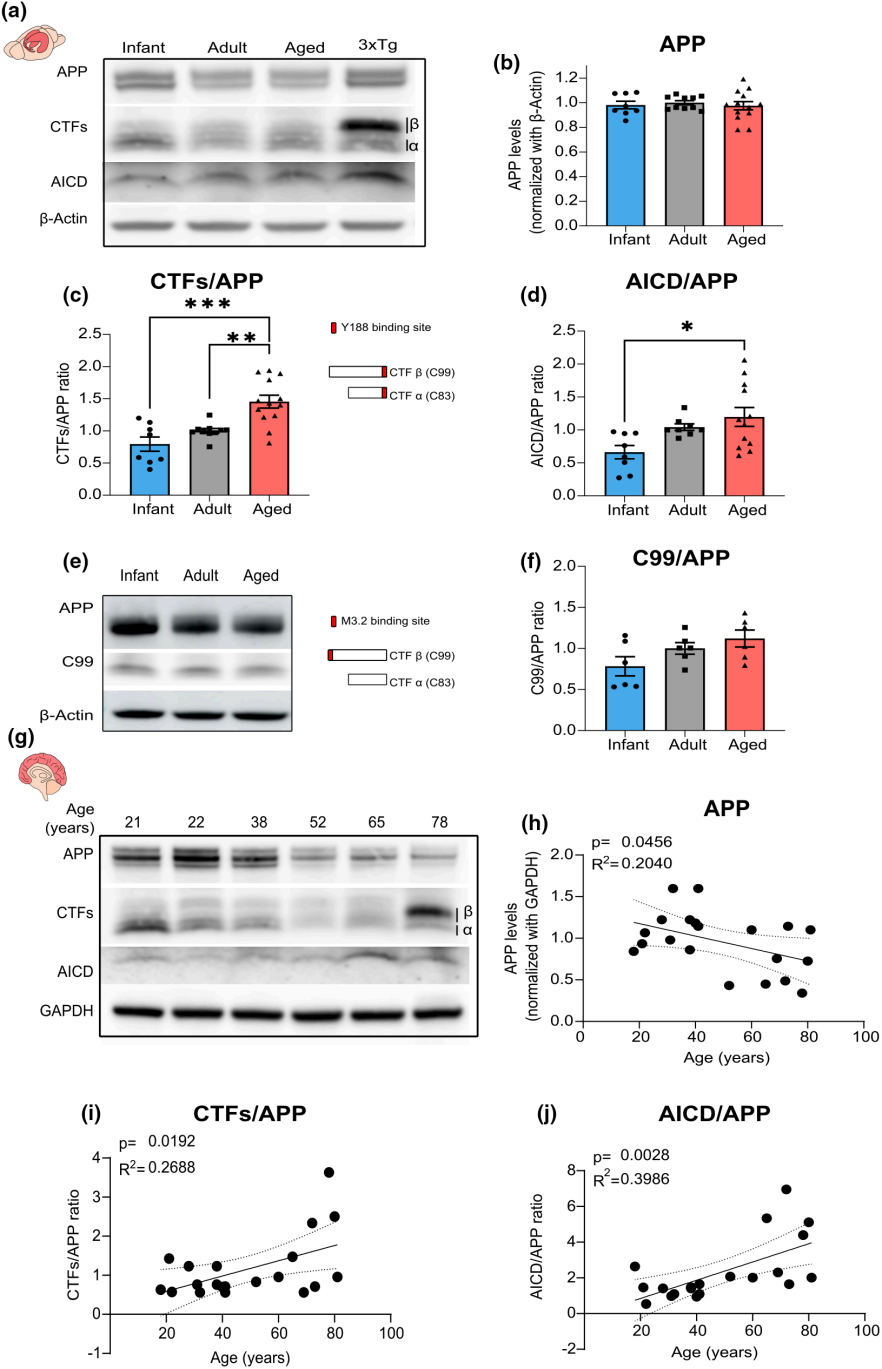
Age is associated with an increase in amyloid precursor protein (APP) processing in mice and humans. (a) Representative Western blot of hippocampal lysates from infant (7–10 days), adult (10–16 weeks), and aged (18–20 months) C57BL/6 wild‐type mice. Membranes were immunoblotted with antibodies for APP C‐terminal (to detect APP full‐length (APP), APP C‐terminal fragments (CTFs β and α), and the APP intracellular domain (AICD)) and β‐actin. A female triple transgenic mouse (3xTg, 6 months) was used as a positive control for APP‐derived fragments. (b) Results from blots shown in a) from mouse hippocampal lysates were normalized with β‐actin and are expressed as the mean ± SEM relative to the adult group (one‐way ANOVA, *p* = 0.8263, *F*(2, 28) = 0.1921, *n* = 8–13). (c) Results from blots shown in a) from mouse hippocampal lysates show the ratio between CTFs and APP are expressed as the mean ± SEM relative to the adult group (Kruskal–Wallis test, *p* = 0.0008, Kruskal–Wallis statistic = 14.21, followed by uncorrected Dunn's test, **p < 0.01, ***p < 0.001, *n* = 8–13). The different CTFs detected with the Y188 antibody (CTFβ (C99) and CTFα (C83)) are schematically represented, with the antibody binding site illustrated in red. (d) Results from blots shown in a) from mouse hippocampal lysates show the ratio between AICD and APP and are expressed as the mean ± SEM relative to the adult group (One‐way ANOVA, *p* = 0.0143, *F*(2, 25) = 5.057, followed by uncorrected Fisher's LSD's multiple comparisons test, *p < 0.05, *n* = 8–13). (e) Representative Western blot of hippocampal lysates from infant, adult, and aged C57BL/6 wild‐type mice. Membranes were immunoblotted with the M3.2 antibody to detect APP and CTFβ (C99) and probed for β‐actin as the loading control. The binding site for the M3.2 antibody is represented in red, showing that it specifically detects CTFβ (C99). (f) Results from blots shown in (e) from mouse hippocampal lysates show the ratio between C99 and APP and are expressed as the mean ± SEM relative to the adult group (one‐way ANOVA, *p* = 0.0802, *F*(2, 15) = 3.000, *n* = 8–13). (g) Representative Western blot of prefrontal cortex human samples (18 to 81 years old). Membranes were immunoblotted with antibodies for APP C‐terminal (to detect APP, CTFs β and α, and AICD) and GAPDH as the loading control. (h) Linear regression graph calculated from blots as shown in (g) shows the variation in APP levels (normalized to GAPDH) depending on the age of human subjects. Statistical analysis was performed using Pearson's correlation (two‐tailed *p*‐value), *p* = 0.0456, R2 = 0.204, *n* = 20. Dotted lines represent the 95% confidence intervals. The values obtained for 20–25‐year‐old subjects were used as reference. (i) Linear regression graph calculated from blots as shown in (g) shows the variation in the ratio between APP‐CTFs and APP depending on the age of human subjects. Statistical analysis was performed using Pearson's correlation (two‐tailed *p*‐value), *p* = 0.0192, R2 = 0.2688, *n* = 20. Dotted lines represent the 95% confidence intervals. The values obtained for 20–25‐year‐old subjects were used as reference. (j) Linear regression graph calculated from blots as shown in (g) shows the variation in the ratio between AICD and APP depending on the age of human subjects. Statistical analysis was performed using Pearson's correlation (two‐tailed *p*‐value), *p* = 0.0028, *R* ^2^ = 0.3986, *n* = 20. Dotted lines represent the 95% confidence intervals. The values obtained for 20–25‐year‐old subjects were used as reference. The full statistical analysis and Western Blot membranes are provided in the Supporting Information.

We thus characterized APP processing throughout time in the hippocampus of wild‐type C57BL/6 mice by detecting APP full‐length (APP), APP C‐terminal fragments (CTFs), and AICD. When compared to adults, aged mice displayed no alterations in the APP levels (Figure 4a,b), but instead, they exhibited an increase in APP processing products. Accordingly, the total CTFs levels in relation to APP, as well as the CTFs absolute values, exhibit an approximately 1.4‐fold increase in aged mice (Figure 4a,c; Figure S4a). We found that the levels of AICD increased with aging, either in relation to APP or in absolute values (Figure 4a,d; Figure S4b).

We performed an approximate discrimination of the CTFs derived from α‐secretase cleavage (CTFα, C83, and ≈9KDa) or β‐secretase processing (CTFβ, C99, and ≈11 KDa) based on their molecular weight and using a triple transgenic mouse (3xTg‐AD) as reference, since this model exhibits a predominant accumulation of CTFβ over the smaller CTFα (Lauritzen et al., 2012; Figure 4a). We found that the CTF β/α ratio increases upon aging (Figure 4a; Figure S4c). A similar tendency was observed for CTFβ (C99) detected with a specific antibody, although not reaching statistical significance (Figure 4e,f; Figure S4d).

To elucidate whether this age‐related pattern of APP processing is also observed in the human brain, we prepared lysates from human postmortem brain tissue (prefrontal cortex) of subjects with different ages (18–81 years old; Figure 4g). We found an inverse correlation between age and APP levels (Figure 4g,h), and a positive correlation in the production of CTFs and AICD from APP (Figure 4g,i,j). This is linked to an increase in the absolute levels of AICD, but not of CTFs (Figure S4e,f) or in the CTF β/α ratio (Figure S4g). We detected a similar pattern of APP levels/processing in samples from AD patients (*n* = 4, 65–81 years old) compared with aged controls (65–89 years old; Figure S4h–k), albeit more variable.

**FIGURE 5:**
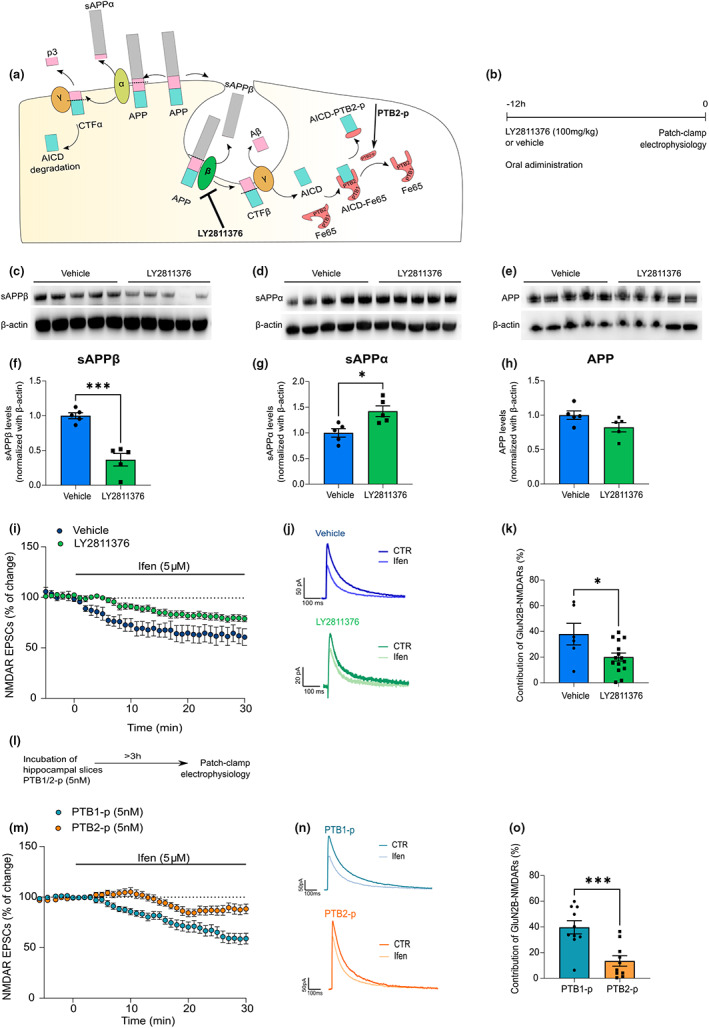
Age‐related increase in βAPP processing contributes to enhanced GluN2B synaptic contribution. (a) Schematic diagram adapted from (Grimm et al., 2013; Müller et al., 2008) showing the experimental approaches to interfere with amyloid precursor protein (APP) processing or AICD signaling. The β‐secretase inhibitor LY2811376 interferes with the APP amyloidogenic processing pathway, but not with the nonamyloidogenic pathway (α‐secretase). The PTB2 peptide (PTB2‐p) interferes with AICD interaction with Fe65 and respective signaling pathways. (b) Representation of the timeline for the β‐secretase inhibitor (LY2811376) treatment in aged mice. (c) Representative Western blot of soluble fractions from hippocampal mouse tissue of aged wild‐type C57BL/6 mice (18–20 months, vehicle vs. LY2811376, 12 h after the treatment). Membranes were immunoblotted with antibodies for sAPPβ and β‐actin as the loading control. (d) Representative Western blot of soluble fractions from hippocampal mouse tissue of aged mice (vehicle vs. LY2811376, 12 h after the treatment). Membranes were immunoblotted with antibodies for sAPPα and β‐actin as the loading control. (e) Representative Western blot of membrane/cytosolic fractions from hippocampal mouse tissue of aged mice (vehicle vs. LY2811376, 12 h after the treatment). Membranes were immunoblotted with antibodies for APP and β‐actin as the loading control. (f) Results from blots of soluble fractions as shown in (c) show sAPPβ levels normalized with β‐actin and are expressed as the mean ± SEM relative to the control condition (vehicle) unpaired *t* test, ***p < 0.001, *n* = 5. (g) Results from blots of soluble fractions as shown in (d) show sAPPα levels normalized with β‐actin and are expressed as the mean ± SEM relative to the control condition (vehicle) unpaired *t* test, *p < 0.05, *n* = 5. (h) Results from blots of membrane/cytosolic fractions as shown in (e) show APP levels normalized with β‐actin and are expressed as the mean ± SEM relative to the control condition (vehicle), unpaired *t* test, *n* = 5. (i) Time course of ifenprodil (5 μM) effect on pharmacologically isolated NMDAR EPSC amplitude in CA1 pyramidal neurons, measured by whole‐cell patch clamp in aged C57BL/6 wild‐type mice (vehicle vs. LY2811376, administration 12 h prior to patch‐clamp recordings). Results are expressed as the mean ± SEM (*n* = 6–15). (j) Traces show NMDAR EPSCs recorded before (CTR) and after 30 min of Ifenprodil 5 μM perfusion (Ifen). (k) GluN2B contribution was calculated as the percentage of change in NMDAR EPSCs after ifenprodil perfusion (for 30 min) in aged C57BL/6 wild‐type mice (vehicle vs. LY2811376). Results are expressed as the mean ± SEM (Unpaired *t* test, *p < 0.05, *n* = 6–15). (l) Representation of the timeline for the incubation of hippocampal slices from aged mice with PTB1‐p or PTB2‐p (5 nM, >3 h before patch‐clamp recordings). (m) Time course of ifenprodil (5 μM) effect on pharmacologically isolated NMDAR EPSC amplitude in CA1 pyramidal neurons, measured by whole‐cell patch clamp in aged C57BL/6 wild‐type mice (PTB1‐p vs. PTB2‐p, incubation for >3 h prior to patch‐clamp recordings). Results are expressed as the mean ± SEM (*n* = 10). (*n*) Traces show NMDAR EPSCs recorded before (CTR) and after 30 min of Ifenprodil 5 μM perfusion (Ifen). (o) GluN2B contribution was calculated as the percentage of change in NMDAR EPSCs after ifenprodil perfusion (for 30 min) in aged C57BL/6 wild‐type mice (PTB1‐p vs. PTB2‐p). Results are expressed as the mean ± SEM (Unpaired *t* test, ***p < 0.001, *n* = 10). The full statistical analysis and Western blot membranes are provided in the Supporting Information.

To test the hypothesis that APP amyloidogenic‐derived fragments are involved in the changes of NMDA function, we treated aged mice with the BACE 1 (BI) inhibitor (LY2811376; 100 mg/kg) to inhibit APP β‐processing (Figure 5a,b). The treated aged mice displayed a significant decrease in GluN2B synaptic contribution (Figure 5i–k) to a magnitude similar to that of untreated adult mice. We confirmed that the treatment was effective in reducing the levels of sAPPβ in the hippocampus (Figure 5c,f). This was accompanied by an increase in the sAPPα levels (Figure 5d,g), while the levels of APP full‐length remained unaltered (Figure 5e,h).

We had previously implicated the Y^682^ENPTY^687^ sequence of AICD on the GluN2B‐mediated effects in adult mice (Pousinha et al. 2017). The PTB2 domain of Fe65 (PTB2‐p) is known to bind to the Y^682^ENPTY^687^ sequence (Feilen et al., 2017), thus interfering with AICD‐Fe65 signaling. In hippocampal slices pre‐incubated with PTB2‐p for >3 h (Figure 5a,l), the aged GluN2B‐NMDAR‐mediated currents were rescued to adult‐like levels (Figure 5m–o; Figure S5a,b), whereas the control PTB1 domain [PTB1‐p; which does not bind either APP or AICD (Cao & Sudhof, 2001)] was devoid of effects, ruling out any nonspecific buffering effects of the PTBs domain.

We concluded that increased APP processing upon aging might lead to exacerbated AICD signaling, thus contributing to the aberrant NMDAR synaptic currents observed in aged cells.

## 3 DISCUSSION

We show that synaptic GluN2B‐NMDARs are regulated by APP in an age‐dependent manner. During postnatal development, APP interacts with GluN2B at the synapse and modulates its synaptic content and evoked currents, possibly having an impact on synaptic maturation. On the contrary, APP‐derived amyloidogenic fragments, namely AICD, contribute to increased GluN2B synaptic contribution upon aging, potentially underlying age‐related synaptic impairments.

We found that the APP interaction regulates the GluN2B‐NMDARs function during development, a life stage when the GluN2B subtype predominates, accounting for more than 50% of NMDAR EPSCs in the CA1 hippocampal region. The electrophysiological output reflected the synaptic NMDAR subunit composition, since infant mice exhibited a high GluN2B/GluN2A ratio in protein levels, according to the previously described NMDAR developmental switch (GluN2B to GluN2A) that occurs during postnatal development (Williams et al., 1993). These data indicate a crucial role for APP at the postsynapse, in contrast with most of the studies so far, which focused on APP function at the presynapse (Marquez‐Sterling et al., 1997, Lyckman et al., 1998), given the low APP levels detected in PSD‐enriched fractions from the adult brain (Kim et al., 1995).

We now found that APP subcellular localization is age‐dependent, since we could observe abundant APP at the postsynapse in infant mice, whereas the levels decline in adulthood and aging, thus suggesting that the role of APP at the synapse shifts with age. Indeed, we proved that the presence of a monoclonal antibody against the APP C‐terminal affects NMDAR‐evoked currents during postnatal development. Importantly, this experimental strategy allows elucidating the role of APP at the postsynapse without interfering with the pre‐synaptic compartment. The fact that preventing the APP β‐processing did not modify this effect further reinforces the direct interaction between APP and NMDAR. It is likely that the kinetics of the action of the antibody is related to an alteration in the synaptic residency of NMDARs, a dynamic process that occurs within minute range (Groc et al., 2006), rather than to the turnover of the NMDAR subunits (half‐life of 2–34 h; Huh & Wenthold, 1999).

The NMDAR‐APP interaction was further investigated in rat hippocampal primary neuronal cultures at immature state (DIV 7–14), since this model recapitulates the synapse development process (Grabrucker et al., 2009) and the NMDAR developmental switch (Corbel et al., 2015), while providing appropriate imaging resolution to study synaptic proteins. Amyloid precursor protein‐depleted neurons exhibited a decreased GluN2B synaptic content, thereby confirming that APP is crucial for NMDAR synaptic levels/activity in immature synapses. Considering the key role of GluN2B currents for synaptic maturation (Gray et al., 2011), we hypothesize that APP‐NMDAR regulation is essential to achieve functional mature synapses. N‐methyl‐D‐aspartate receptor activity has been associated with PSD‐95 recruitment to new synapses (de Roo et al., 2008). This is consistent with our observations in APP‐depleted neurons, in which the decreased GluN2B synaptic content is accompanied by a reduction in PSD‐95 dendritic area and cluster size. However, we cannot discard the possibility that APP could directly regulate PSD‐95 area and average particle size. In that case, the decline in GluN2B synaptic content might occur as an indirect response. The results obtained upon APP C‐terminal disruption in infant mice show that it is possible to rapidly interfere with APP‐NMDAR regulation, favoring the hypothesis of NMDAR synaptic stabilization rather than a structural role in PSD‐95.

These data might seem contradictory with the observation that the APP KO model only presents synaptic deficits after 10 months of age (Dawson et al., 1999). This may result from the functional redundancy of APP and APP‐like proteins (APLP1 and ALPL2). In accordance, constitutive triple knockout mice (TKO) die after birth (Heber et al., 2000), whereas Nex‐Cre cTKO (conditional triple KO in excitatory forebrain neurons starting during embryonic development) present gross brain morphology alterations (Steubler et al., 2021), showing a crucial role for APP family members during development. Our combination of in vitro silencing and acute interference of the APP C‐terminal domain ex vivo overcome a possible compensation by APP family members, while allowing to study APP specifically during postnatal developmental stages.

Considering that the antibody against the APP C‐terminal had a significant effect on NMDAR‐mediated currents in infant mice, this seems to be the domain responsible for interacting and regulating NMDARs at this stage. In particular, the YENPTY motif is highly conserved in APP family members and different species (Shariati & de Strooper, 2013) and known to interact with several proteins, such as Fe65 (van der Kant & Goldstein, 2015), possibly acting, directly or indirectly, as the interacting site for NMDARs. We had previously shown that neurons expressing a mutated form of AICD in the YENPTY motif lose their ability to modulate NMDA currents in the adult rat (Pousinha et al., 2017). Accordingly, mouse models in which the APP C‐terminal domain is mutated (Matrone et al., 2012) or depleted (APPdCT15 knockin mice/APLP2 KO; Klevanski et al., 2015) show high postnatal lethality, impairments in synaptic plasticity, and memory in adult stages, which might be explained by NMDAR dysregulation, although this hypothesis has not been explored so far.

We could not affect NMDAR EPSCs in adult/aged animals when interfering with the APP C‐terminal domain in a 60 min time window. Considering the technical approach we used, it is possible that the accessibility of the APP C‐terminal epitope at the postsynapse might be precluded in adult stages, due to the extensive alterations that occur in PSD structure and composition after postnatal development (Gonzalez‐Lozano et al., 2016). However, we can also postulate that the APP regulation of NMDARs has indeed a higher impact during development, which is consistent with the marked decline of postsynaptic APP levels observed in adult stages. Moreover, it is possible that the APP‐NMDAR regulation mainly occurs through GluN2B, whose synaptic contribution declines after development. Our observation of a reduced effect of ifenprodil on the neurons from infant mice exposed to the APP C‐terminal antibody indicates that GluN2B‐NMDARs are highly affected by disruption of APP C‐terminal. Since APP interacts with both GluN2B and GluN2A in adult stages, it is extremely difficult to determine whether there is a subunit preferential binding, especially considering that tri‐heteromeric complexes (GluN1/GluN2A/GluN2B) also exist in the hippocampus (Rauner & Köhr, 2011). Given the increased association of PSD‐95 with NMDARs in mature synapses (Elias et al., 2008), we can postulate that the NMDAR synaptic clustering becomes APP‐independent upon adulthood. However, the fact that the Camk2a‐Cre cTKO mouse model (triple conditional knockout for APP family members in excitatory forebrain neurons starting at postnatal weeks 3–4) exhibits LTP impairments and reduced NMDAR‐mediated responses in adult stages suggests that some form of NMDAR regulation by APP family members still occurs later in life (Lee et al., 2020).

We found that NMDAR deactivation kinetics become slower upon aging, possibly explaining the increased duration of NMDAR field excitatory postsynaptic potentials (fEPSPs) described previously in aged rodents (Jouvenceau et al., 1998). These data correlate with the augmented GluN2B contribution in aged mice, possibly reflecting the higher GluN2B synaptic retention reported in previous studies (al Abed et al., 2020; Zamzow et al., 2013). We postulate that APP contributes to these alterations, controlling NMDAR function in aged synapses. This hypothesis is supported by the synaptic plasticity and learning/memory deficits observed in APP KO‐aged mice, but not at earlier stages (10 months vs. 4 months; Dawson et al., 1999).

Our findings suggest that the APP‐NMDAR regulation in aged synapses occurs through APP‐derived fragments rather than through the full‐length protein. We detected a significant enhancement of APP processing into CTFs and AICD upon aging. Importantly, we observed the same profile in human brain samples, in which we established a positive correlation between APP processing and aging. Considering the previously described accumulation of CTFs (Burrinha et al., 2021) and increased BACE1 activity (Fukumoto et al., 2004) in aged mice, we postulate that this accumulation of APP‐derived amyloidogenic fragments will eventually lead to alterations in NMDAR function. To test this hypothesis, we used a β‐secretase 1 (BACE 1) inhibitor in aged animals to block the APP amyloidogenic processing, which resulted in a decrease in GluN2B synaptic contribution of about 20%, a magnitude closer to that obtained in adult mice.

Although we did not single out the APP amyloidogenic fragment responsible for this effect, the AICD has emerged as a potential target. This APP‐derived fragment is affected by BACE1 inhibition, since the biologically active AICD form mainly derives from the amyloidogenic pathway (Goodger et al., 2009). We have previously shown that the overexpression of AICD in adult synapses leads to a NMDAR‐GluN2B electrophysiological profile similar to the one we now report in aging (Pousinha et al., 2017). Concomitantly, we detect an increase in AICD levels upon aging. The fact that interfering with AICD‐Fe65 interaction rescued the GluN2B contribution, further strengthens a role for AICD in the observed effects. Accordingly, the disruption of the AICD‐Fe65 interaction normalized the GluN2B contribution to adult‐like levels. The mechanism by which PTB2‐p affects GluN2B‐NMDAR‐mediated currents may depend on direct binding of PTB2 to AICD after uptake in an endocytosis‐dependent manner, as described for different cytosolic proteins, such as tau and alpha‐synuclein (Peng et al., 2020) or by direct transmission over the membrane, as postulated for monomeric alpha‐synuclein (Lee et al., 2008) or proteins carrying specific transmission sequences that resemble positively charged nuclear localization sequences (Suzuki, 2012). The AICD‐Fe65 complex induces multiple signaling pathways (Augustin & Kins, 2021), as well as transcriptional activity in several target genes, including the one encoding for GluN2B (Grimm et al., 2013; Pousinha et al., 2017). The fact that we did not detect alterations in the synaptic levels of GluN2B by aging suggests that other transcriptional targets may be involved in the observed effects, namely kinases/phosphatases that are known to regulate GluN2B phosphorylation status, Ca^2+^ permeability, or trafficking (Murphy et al., 2014).

Therefore, our findings could help to clarify an apparent paradox in the field: Although total NMDAR levels are known to decline in physiological aging and AD (Jacob et al., 2007; Magnusson, 2012), NMDAR antagonists such as memantine are effective in counteracting cognitive decline and approved for clinical use in AD (Reisberg et al., 2003). We show that the NMDARs that remain at aged synapses have altered properties from adulthood, staying open for longer times and being more dependent on the GluN2B subtype. An imbalance in subunit contribution toward GluN2B is expected to decrease the threshold for LTP (Yashiro & Philpot, 2008), possibly contributing to the LTD‐LTP shift reported by our group in aged rats (Temido‐Ferreira et al., 2020). Therefore, the increased contribution of GluN2B may lead to synaptic dysregulation in physiological aging, while increasing the susceptibility to neurodegeneration, in which GluN2B‐NMDARs become overactivated (Li et al., 2011). This mechanism might be particularly relevant in AD, since the pathological accumulation of APP amyloidogenic fragments is expected to further enhance GluN2B contribution. More importantly, the now unraveled mechanism may explain the protective effect of the APP A6737 Icelandic variant in age‐related cognitive decline and AD. This mutation decreases the affinity of APP for BACE1, reducing its cleavage activity by 40% (Jonsson et al., 2012), and possibly reducing the herein described GluN2B aberrant synaptic contribution.

In conclusion, we describe two alternative mechanisms by which APP controls GluN2B‐NMDARs, depending on the age. We have discovered a new physiological role for APP at the postsynapse, being essential to maintain GluN2B synaptic content/currents in immature synapses. Moreover, we show that the age‐related increase in APP processing contributes to a higher GluN2B synaptic contribution. While the first mechanism might play a crucial role in synaptic maturation, the latter could be involved in age‐associated synaptic dysfunction.

## 4 METHODS

### 4.1 Human samples

The use of human samples was conducted in accordance with the Helsinki Declaration and National Ethical Guidelines. Protocols were approved by the Local Ethics Committee and the National Data Protection Committee. Samples of postmortem brain tissue from the prefrontal cortex were obtained from the National Institute of Legal Medicine and Forensic Sciences, Coimbra, Portugal, the Neuro‐CEB Biological Resource Center (BRC), France, and the Newcastle Brain Tissue Resource, United Kingdom. Samples from human AD patients correspond to Braak Stage VI. The information about the gender and post‐mortem delay is indicated in the Supporting Information.

### 4.2 Animals

Male and female wild‐type C57BL/6 mice at different ages were used: infant (7–10 days), adult (10–16 weeks), and aged (18–20 months). A C7BL/6‐129SvJ female mouse bearing three mutations (3xTg‐AD) associated with familial AD (amyloid precursor protein [APPswe], presenilin‐1 [PSEN1], and microtubule‐associated protein tau [MAPT]; Mutant Mouse Research and Resource Center at The Jackson Laboratory) was used as control. The detailed procedures are described in the Supporting Information.

### 4.3 BACE1 inhibitor

LY2811376 was obtained from Medchem Express (Sweden) and prepared in 10% DMSO, 40% PEG300, 5% Tween‐80, and 45% saline. C57BL/6 infant (7–10 days) or aged mice (18–20 months) received LY2811376 at 100 mg/kg body weight by sucking reflex or oral gavage, respectively, as described in Filser et al. (2015). Animals treated with LY2811376 (infant: *n* = 6; aged: *n* = 5) or vehicle (infant: *n* = 3; aged: *n* = 5) were sacrificed ≈12 h after treatment.

### 4.4 Expression and purification of His‐tagged fusion proteins (PTB1‐p and PTB2‐p) in *Escherichia coli*

The recombinant fusion proteins were expressed in Escherichia coli BL21 (DE3) RIL cells transformed with the plasmid pET21d Fe65‐PTB2 6xHis (Radzimanowski, Beyreuther et al., 2008) or pET24d Fe65‐PTB1 6xHis (Radzimanowski, Ravaud et al., 2008). Transformed bacteria were cultivated at 37°C in 2xYT media containing 100 mg/L ampicillin (for Fe65‐PTB2 6xHis) or 30 mg/L kanamycin (for Fe65‐PTB1 6xHis). Twenty hours after inducing the protein expression via 1 mM IPTG (Isopropyl β‐D‐1‐thiogalactopyranoside), the bacteria were sonicated five times with 10 pulses (Sonoplus HD 2200 sonicator, Bandelin) in lysis buffer (50 mM Tris/300 mM NaCl/10 mM Imidazole/pH 8), with EDTA‐free protease inhibitor and 1 mM DTT. The lysate was centrifuged for 45 min at 11,300 *g* at 4°C. The proteins were purified using an Äkta Purifier 10 system (Cytiva) with a His‐Trap HP column (GE Healthcare). After intense washing, the bound proteins were eluted in 50 mM Tris/300 mM NaCl/300 mM imidazole/pH 8. Using a PD‐10 desalting column (Cytiva), the elution buffer was exchanged to HEPES buffer (20 mM HEPES/150 mM NaCl/pH 7.2).

### 4.5 Patch‐clamp electrophysiology

The whole‐cell patch‐clamp electrophysiology recordings to measure pharmacologically isolated evoked‐NMDAR EPSCs were performed as described previously by our group (Pousinha et al. 2017). The detailed protocol is described in the Supporting Information.

For the analysis of NMDAR EPSCs deactivation kinetics, decay time was fitted with a double exponential function, using Clampfit software, to calculate both slow and fast decay time constants, τ_fast_ and τ_slow_, respectively. The weighted time constant (τ_weighted_) was calculated using the relative contribution from each of these components, applying the formula: *τ*_w_ = [(*a*_f_. *τ*_f_) + (*a*_s_. *τ*_s_)]/(*a*_f_ + *a*_s_), where *a*_f_ and *a*_s_ are the relative amplitudes of the two exponential components, and *τ*_f_ and *τ*_s_ are the corresponding time constants.

To calculate GluN2B‐NMDAR contribution, NMDAR EPSCs were measured immediately before (5 min) and 25–20 min after ifenprodil (5 μM) perfusion to selectively block GluN2B‐NMDARs.

To interfere with the APP‐NMDAR interaction, the APP C‐terminal antibody Y188 (ab32136, Abcam) was added to the intracellular solution to a final concentration of 0.4 μg/ml (2.57 nM). In the control condition, the same antibody was heat‐inactivated by incubation at 98°C for 10 min. The percentage of reduction in NMDAR EPSCs due to the APP C‐terminal antibody incubation was calculated comparing the baseline amplitude (15–20 min) with the final amplitude (55–60 min) and normalized with the control condition for each age.

To interfere with the AICD‐Fe65, hippocampal slices from aged mice were preincubated prior to recording as indicated in each figure with either the PTB2‐p or the respective control (PTB1‐p) at a final concentration of 5 nM.

### 4.6 Protein analysis

#### 4.6.1 Fractionation into PSD‐enriched fractions

The fractionation protocol was adapted from Frandemiche et al. (2014). The integrity of non‐PSD was verified by immunoblotting for synaptophysin, which was enriched in the non‐PSD fraction, and the integrity of the PSD fraction was confirmed by the immunoblotting of PSD‐95 enriched in this compartment. The detailed protocol is described in the Supporting Information.

#### 4.6.2 Synaptosomes preparation

The protocol for synaptosome preparation was adapted from Lopes et al. (1999). The detailed protocol is described in the Supporting Information.

#### 4.6.3 Extraction of soluble and membrane/cytosolic proteins

Soluble proteins were extracted from mouse hippocampal tissue with DEA buffer, whereas membrane/cytosolic proteins were extracted with RIPA buffer, as described in Willem et al. (2015). The detailed protocol is described in the Supporting Information.

#### 4.6.4 Co‐immunoprecipitation

Mouse hippocampal or human postmortem cortical tissue was resuspended in immunoprecipitation buffer (50 mM Tris HCl pH 7.5; 150 mM NaCl; 2 mM EDTA; 1% Triton with protease and phosphatase inhibitors). The immunoprecipitation protocol was adapted from Tomé et al. (2021) using Dynabeads Protein A (10001D, Invitrogen), and the detailed protocol is described in the Supporting Information. At the end of the experiment, the immunoprecipitated samples were gently resuspended in 60 μl of preheated 2× sample buffer (140 mM Tris HCl pH 6.8, 4% SDS, 13.6% glycerol, 272 mM DTT, 0.004% Blue bromophenol) in RIPA (50 mM Tris, 1 mM EDTA, 150 mM NaCl, 0.1% SDS, 1% Tergitol‐type NP‐40, pH 8.0, and incubated for 10 min at 95°C). The supernatant was collected and used for Western blot analysis.

#### 4.6.5 Western blotting

Mouse and human frozen tissue samples were resuspended in A‐EDTA buffer (10 mM HEPES‐KOH pH 7.9, 10 mM KCl, 1.5 mM MgCl_2_, 0.1 mM EDTA, and 0.3% NP‐40 with protease and phosphatase inhibitors), as described in Pousinha et al. (2017).

For APP, APP‐CTFs, and AICD analysis, optimal conditions for low molecular mass protein separation were used, as described in Willem et al. (2015). Proteins were separated using precast gradient Tricine Protein Gels (10–20%, 1 mm, Novex) in Tris‐tricine buffer (1 M Tris, 1 M Tricine, 1% SDS). Samples were electrotransferred at 400 mA for 1 h to 0.2 μm nitrocellulose membranes using a Tris‐glycine buffer (25 mM Tris, 190 mM glycine) with 20% ethanol. Proteins transferred to nitrocellulose membranes were additionally denatured by boiling the membrane in PBS for 5 min, acting as an antigen retrieval step to detect AICD, as described in Pimplikar & Suryanarayana (2011).

For the remaining proteins, electrophoresis was performed in Tris‐glycine buffer with 10% SDS using 10–12% and 4% acrylamide resolving and stacking gels, respectively. Proteins were electrotransferred to 0.45 μm polyvinylidene fluoride (PVDF) membranes in Tris‐glycine buffer with 20% methanol at 350 mA for 90 min.

After transfer, membranes were blocked with 3% BSA in TBS‐T (20 mM Tris, 150 mM NaCl, 0.1% Tween‐20) at room temperature (RT) for 1 h and incubated with primary antibodies (diluted in 3% BSA TBS‐T) overnight at 4°C. After three washing steps with TBS‐T, membranes were incubated with horseradish peroxidase‐conjugated anti‐mouse or anti‐rabbit secondary antibodies for 1 h at RT. After three washing steps with TBS‐T, chemiluminescent detection was performed with enhanced chemiluminescence (ECL) Western blotting detection reagent (GE Healthcare). For AICD detection, longer exposure times were applied.

For the analysis of soluble APP fragments (sAPPβ and α), DEA fractions were loaded in the gel, followed by electrophoresis in Tris‐glycine buffer with 10% SDS. Following transfer, membranes were blocked in I‐Block solution (1 g Topix I‐Block, Thermo Fischer Scientific, in 500 ml PBS, 0.2% Tween 20) for 1 h at RT and incubated with primary antibodies diluted in I‐Block solution overnight at 4°C. The washing steps were performed with PBS‐Tween buffer and the secondary antibodies were diluted in I‐Block solution and incubated for 1 h at RT. For ECL detection, membranes were incubated for 1 min at RT with peroxidase substrate (Western lightning ultra, PerkinElmer) and signals were captured with phospho‐Fusion imager, Vilber Lourmat.

Optical density was determined with ImageJ, according to the software instructions (Ferreira & Rasband, 2012). The detailed protocols, as well as the full‐length blots with the molecular weight standards (NZYColour Protein Marker I, NZYTech), are provided in the Supporting information.

#### 4.6.6 Primary neuronal cultures

Hippocampal neurons were cultured from 18‐day Sprague–Dawley rat embryos, adapting the protocol from (Faria‐Pereira et al., 2022). Cells were plated on poly‐D‐lysine‐coated glass coverslips in 24‐multiwell plates at a final density of 70,000 cells/coverslip, in neuronal plating media ((Minimum Essential Medium) supplemented with 10% horse serum, 0.6% glucose, and 100 U/ml Pen‐Strep) and maintained at 37°C in a 5% CO_2_‐humidified incubator. After 4 hours, the plating medium was replaced for neuronal culture medium: Neurobasal medium (Gibco–Life Technologies) supplemented with B‐27 supplement, 25 μM Glutamic acid, 0.5 mM Glutamine, and 20 U/ml penicillin/streptomycin. Cultures were maintained in the humidified incubator for 2 weeks, feeding the cells once per week with neuronal culture medium by replacing half of the medium per well. This protocol is associated with an enrichment in neuronal cells (NeuN‐positive cells ≈80%) and a low number of glial cells (GFAP‐positive cells ≈6%) at DIVs 11–14 (Faria‐Pereira et al. 2022).

The detailed protocol is described in the Supporting Information.

#### 4.6.7 Neuronal transfection

Primary neuronal cultures were transiently transfected at DIV 7‐8 using the calcium phosphate transfection protocol adapted from Silva et al. (2019); Jiang et al. (2004). The detailed protocol is described in the Supporting Information.

#### 4.6.8 Plasmid generation

Primary neuronal cultures were transfected with AAV‐shRNA–mCherry plasmids, with a shRNA against APP or a shRNA control sequence.

The control plasmid was kindly provided by Dirk Grimm (University of Heidelberg) and corresponds to AAV‐U6‐NS1‐CMV‐mCherry plasmid, where NS1 is a nonsilencing sequence: GTAACGACGCGACGACGTAA, with no identified targets in the rat genome, confirmed by NCBI Basic Local Alignment Search Tool (BLAST).

For the generation of the shRNA‐APP construct, we used the following sequence: GCACTAACTTGCACGACTATG (Young‐Pearse et al., 2007), which is complementary to the mRNA NCBI reference sequence for rat APP (Rattus norvegicus amyloid beta precursor protein (App), NM_019288.2). This construct, which was provided by Tracy Young‐Pearse (Harvard Medical School) in the pENTR‐U6 vector, was then subcloned into an adeno‐associated virus backbone (AAV‐U6‐shRNA empty‐CMV‐mCherry plasmid), kindly provided by Dirk Grimm, as described in the Supporting Information. Both plasmids were purified using the EndoFree Plasmid Maxi Kit (Qiagen) and verified by DNA sequencing (Primer 5′‐GGGCCTATTTCCCATGATTCC‐3′).

#### 4.6.9 Immunocytochemistry

The immunostaining protocol was adapted from Ferreira et al. (2017), as described in the Supporting Information.

#### 4.6.10 Microscopy imaging and analysis

All images were acquired in a Zeiss LSM 880 laser scanning confocal microscope using a Plan‐Apochromat 63×/1.4 oil immersion objective.

For the analysis of APP immunofluorescence in transfected neurons, the APP (Alexa Fluor 488) relative fluorescence intensity was manually quantified using ImageJ, after maximum intensity projection. For each condition, seven transfected neurons were analyzed by defining regions of interest (ROI), which corresponded to the cell bodies using the mCherry channel. The average intensity of Alexa Fluor 488 was then determined for each ROI. All values were normalized to the average intensity in transfected neurons from the control condition (%).

For the analysis of GluN2B/PSD95 in dendrites of transfected neurons, the analysis of GluN2B/PSD95 in transfected neurons was performed using an in‐house‐developed macro for ImageJ.

### 4.7 Statistical analysis

All statistical analyses were performed with GraphPad Prism software. Results are referred in the text as mean ± standard error of the mean (SEM), which is also represented in the graphs, together with dot blots with individual values. Statistical analyses were performed after evaluating normality using the Shapiro–Wilk test. When the distribution was normal in all groups, the statistical comparison included unpaired *t* test or one‐way ANOVA followed by uncorrected Fisher's LSD's multiple comparisons test. When the distribution was not normal, the statistical comparison was performed by Mann–Whitney test or Kruskal–Wallis followed by uncorrected Dunn's multiple comparisons test. When the graphs represent relative levels, the values are expressed in relation to the reference group. Correlations between parameters were determined according to Pearson's correlation coefficient. Significance was determined according to the following criteria: *p* > 0.05 = not significant, *p < 0.05, **p < 0.01, ***p < 0.001, and ****p < 0.0001. The complete statistical analysis is provided in Supporting information.

## AUTHOR CONTRIBUTIONS

JR‐S has written the draft and performed the fractionation, co‐immunoprecipitation, Western blotting, immunocytochemistry assays, and the primary neuronal cultures, assisted by MT‐F and JEC. PAP performed the whole‐cell patch‐clamp recordings. FJE provided the tools to study APP‐NMDAR interaction. MW provided tools to perform Western blot analysis of APP processing. LVL assisted with the analysis of Western blot of APP processing. HM and JR‐S designed and cloned the shAPP plasmids. SKö expressed and purified the PTB1‐p and PTB2‐p under the supervision of SKi. AR and SM performed the in vivo pharmacological treatment of infant and aged mice, JD performed the respective fractionation and WB of APP processing and AR performed whole‐cell patch‐clamp recordings of treated infant mice. JRS, LVL, and PAP designed the experiments and wrote the manuscript. LVL and PAP coordinated the project. All authors revised the manuscript and discussed the experimental findings.

## CONFLICT OF INTEREST

All the authors declare no known conflicts of interest associated with this publication, and there has been no significant financial support for this work that could have influenced its outcome. The manuscript has been read and approved by all named authors.

## Supporting information


Appendix S1.

## Data Availability

All the software used to data analysis is commercially available, and the respective information is provided in each respective section. The data that support the findings of this study are available from the corresponding authors upon request.
