# Supplementary material for: Age‐dependent NMDA receptor function is regulated by the amyloid precursor protein
Source: Aging Cell. 2023 Jan 26;22(3):e13778. doi: 10.1111/acel.13778 (PMC10014064; doi:10.1111/acel.13778)

Supporting Information ACE-22-0554R2

This document includes:

1) Supplementary Figures and Legends
2) Detailed description of Materials and Methods
3) Supplementary Table 1 – Information on human samples
4) Supplementary Table 2 - Complete statistical analysis
5) Full-length blots with the molecular weight standards (NZYColour Protein Marker I, NZYTech).

Fig. S1

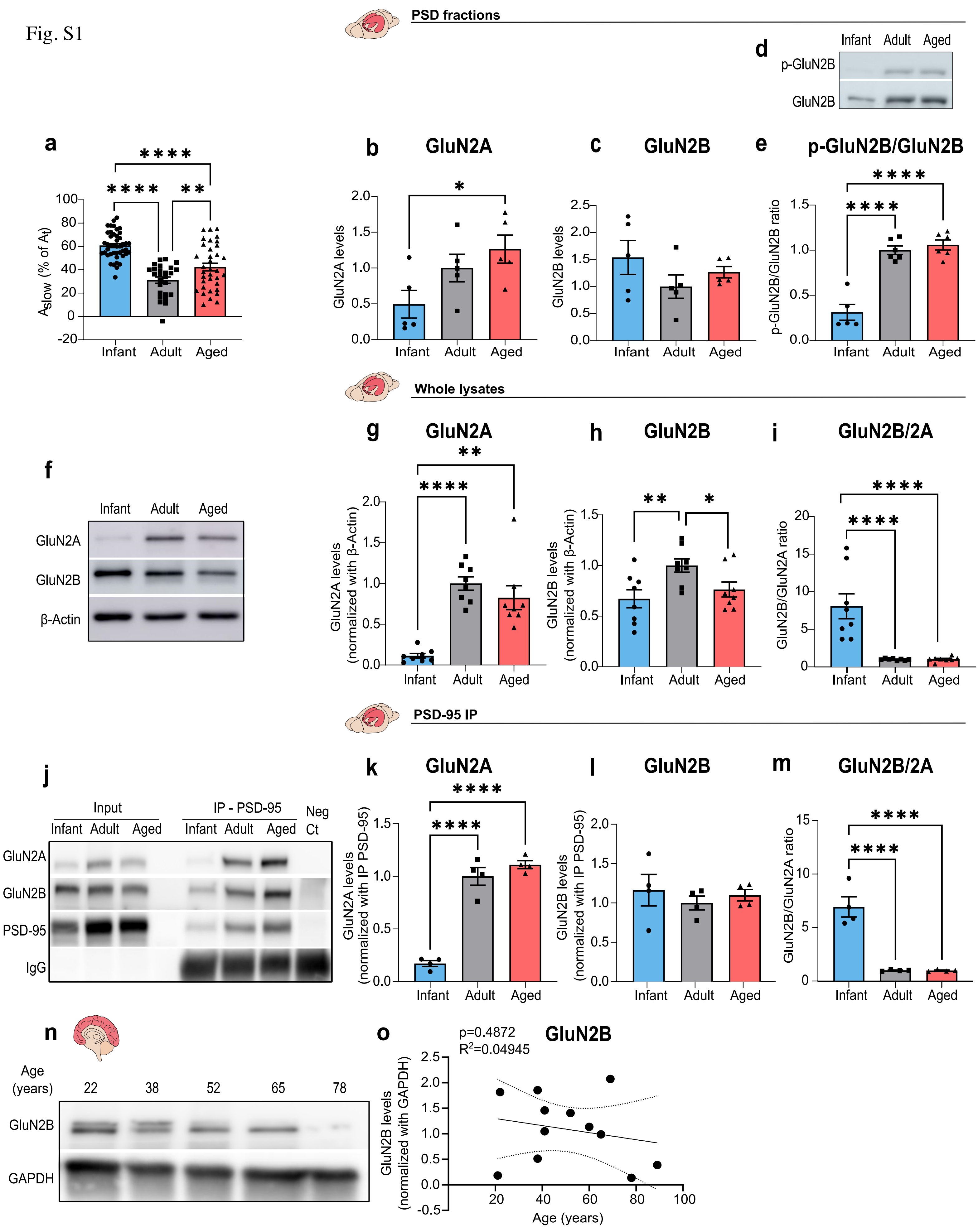
PSD fractions
Whole lysates
PSD-95 IP
a
b GluN2A
c GluN2B
d
e p-GluN2B/GluN2B
f
g GluN2A
h GluN2B
i GluN2B/2A
j
k GluN2A
l GluN2B
m GluN2B/2A
n
o GluN2B
Infant Adult Aged
p-GluN2B
GluN2B
GluN2A
β-Actin
Input
IP - PSD-95
Neg Ct
PSD-95
IgG
Aslow (% of At)
GluN2A levels
GluN2B levels
p-GluN2B/GluN2B ratio
GluN2A levels (normalized with β-Actin)
GluN2B levels (normalized with β-Actin)
GluN2B/GluN2A ratio
GluN2A levels (normalized with IP PSD-95)
GluN2B levels (normalized with IP PSD-95)
Age (years)
22 38 52 65 78
GAPDH
p=0.4872
R²=0.04945
GluN2B levels (normalized with GAPDH)

Supplementary Figure 1 – GluN2B synaptic current component and levels in mouse hippocampus and human brain.

(a) A_{slow} was calculated as the amplitude of the slow component of NMDAR EPSCs normalized to the total amplitude (%), measured by whole-cell patch-clamp recordings in CA1 hippocampal neurons from infant (7-10 days), adult (10 – 16 weeks) and aged (18 – 20 months) C57BL/6 mice. Results are expressed as the mean ± SEM (One-way ANOVA, $p<0.0001$, $F(2, 102)=34.58$, followed by Uncorrected Fisher's LSD's multiple comparisons test, **$p<0.01$, ****$p<0.0001$, n=26-44).

(b, c) Results from blots as shown in Figure 1h) from PSD-enriched fractions from the hippocampal tissue of infant, adult and aged mice. GluN2B and GluN2A levels (not normalized for PSD-95) are expressed as the mean ± SEM relative to the adult group. b) One-way ANOVA, $p=0.0444$, $F(2, 12)= 4.084$, followed by Uncorrected Fisher's LSD's multiple comparisons test *$p<0.05$, n=5. c) One-way ANOVA, $p=0.2822$, $F (2, 12) = 1.408$, n=5.

(d) Representative western blot of PSD-enriched fractions from the hippocampal tissue of infant, adult and aged mice. Membranes were immunoblotted with antibodies for p-GluN2B (Y1472) and GluN2B.

(e) Results from blots as shown in d) correspond to the p-GluN2B/GluN2B ratio relative to the adult group and are expressed as the mean ± SEM (One-way ANOVA, $p<0.0001$, $F(2, 14)=41.04$, followed by Uncorrected Fisher's LSD's multiple comparisons test, ****$p<0.0001$, n=5-6).

(f) Representative western of hippocampal lysates from infant, adult and aged C57BL/6 wild-type mice. Membranes were immunoblotted with antibodies for GluN2A, GluN2B and β-actin.

(g, h) Results from blots as shown in f) from hippocampal lysates were normalized with β-actin and are expressed as the mean ± SEM relative to the adult group. g) Kruskal Wallis test, $p=0.0002$, Kruskal-Wallis statistic=16.81, followed by Uncorrected Dunn's test, **$p<0.01$, ****$p<0.0001$, n=8; h) One-way ANOVA, $p=0.019$, $F(2, 21)=4.813$, followed by Uncorrected Fisher's LSD's multiple comparisons test, **$p<0.01$, *$p<0.05$, n=8).

(i) Results from blots as shown in f) from hippocampal lysates show the GluN2B/GluN2A ratio and are expressed as the mean ± SEM relative to the adult group (One-way ANOVA, $p<0.0001$, $F(2, 21)=18.00$, followed by Uncorrected Fisher's LSD's multiple comparisons, ****$p<0.0001$, $n=8$).

(j) Representative western blot of hippocampal lysates from infant, adult and aged C57BL/6 wild-type mice immunoprecipitated for PSD-95. Membranes were immunoblotted with antibodies for GluN2A, GluN2B and PSD-95.

(k, l) Results from blots as shown in j) from PSD-95 immunoprecipitated samples were normalized with PSD-95 and are expressed as the mean ± SEM relative to the adult group. k) One-way ANOVA, $p<0.0001$, $F(2, 9)=85.28$, followed by Uncorrected Fisher's LSD's multiple comparisons test, ****$p<0.001$, $n=4$. l) One-way ANOVA, $p=0.6946$, $F(2, 9)= 0.3796$, $n=4$).

(m) Results from PSD-95 immunoprecipitated samples show the GluN2B/GluN2A ratio expressed as the mean ± SEM relative to the adult group (One-way ANOVA, $p<0.0001$, $F(2, 9)=39.56$, followed by Uncorrected Fisher's LSD's multiple comparisons test, ****$p<0.0001$, $n=4$).

(n) Representative western blot of prefrontal cortex human samples (21 to 89 years old). Membranes were immunoblotted with antibodies for GluN2B and GAPDH.

(o) Linear regression graph calculated from blots as shown in n) shows the variation in GluN2B levels (normalized with GAPDH) depending on the age of human subjects ($n=12$). Statistical analysis was performed using Pearson's correlation (two-tailed p value), $p=0.4872$, $R^2=0.04945$. Dotted lines represent the 95% confidence intervals. The values obtained for 20–25-year-old subjects were used as reference.

The full statistical analysis and Western Blot membranes are provided in the Supporting Information.

Fig. S2

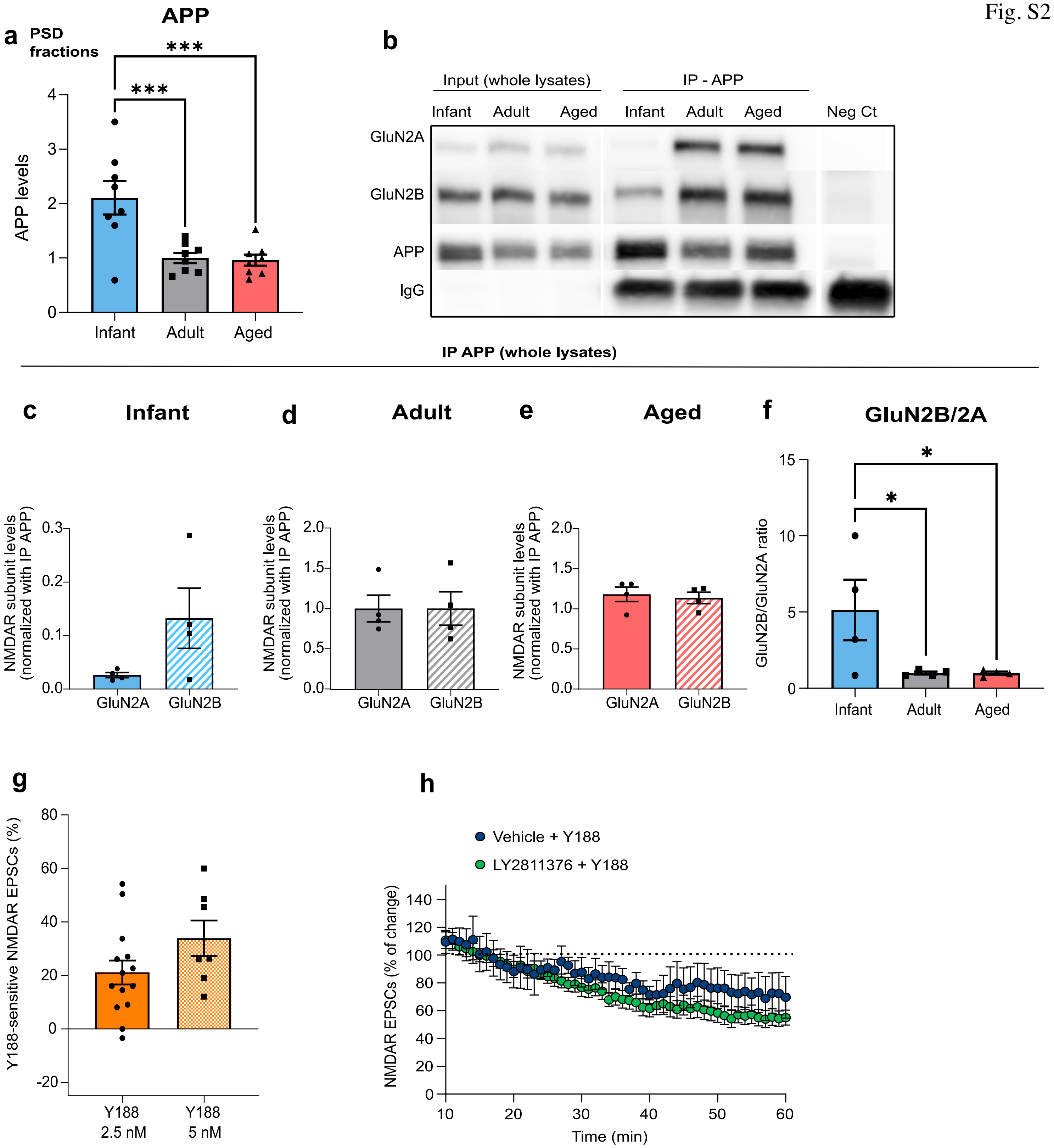

a
PSD fractions
APP
***
***
APP levels
0
1
2
3
4
Infant
Adult
Aged
b
Input (whole lysates)
IP - APP
Infant
Adult
Aged
Infant
Adult
Aged
Neg Ct
GluN2A
GluN2B
APP
IgG
IP APP (whole lysates)
c
Infant
NMDAR subunit levels (normalized with IP APP)
0.0
0.1
0.2
0.3
GluN2A
GluN2B
d
Adult
NMDAR subunit levels (normalized with IP APP)
0.0
0.5
1.0
1.5
2.0
GluN2A
GluN2B
e
Aged
NMDAR subunit levels (normalized with IP APP)
0.0
0.5
1.0
1.5
2.0
GluN2A
GluN2B
f
GluN2B/2A
*
*
GluN2B/GluN2A ratio
0
5
10
15
Infant
Adult
Aged
g
Y188-sensitive NMDAR EPSCs (%)
-20
0
20
40
60
80
Y188 2.5 nM
Y188 5 nM
h
Vehicle + Y188
LY2811376 + Y188
NMDAR EPSCs (% of change)
0
20
40
60
80
100
120
140
10
20
30
40
50
60
Time (min)

Supplementary Figure 2 – Synaptic APP expression and co-immunoprecipitation with NMDARs. Electrophysiological controls of APP-NMDA interaction.

(a) Results from blots as shown in Figure 2a) from PSD-enriched fractions from the hippocampal tissue of infant (7-10 days), adult (10 – 16 weeks) and aged (18 – 20 months) mice. APP levels (not normalized for PSD-95) are expressed as the mean ± SEM relative to the adult group (One-way ANOVA, $p=0.0005$, $F(2, 21)=11.19$, followed by Uncorrected Fisher's LSD's multiple comparisons test, ***$p<0.001$, $n=8$).

(b) Representative western blot of whole lysates from the hippocampi of infant, adult and aged wild-type C57BL/6 mice immunoprecipitated for APP. Membranes were immunoblotted with antibodies for GluN2A, GluN2B and APP.

(c, d, e) Results show the levels of GluN2A and GluN2B in APP immunoprecipitates from infant, adult and aged mice, in blots as shown in b) and are expressed as the mean ± SEM, normalized with immunoprecipitated APP, and relative to the adult group.

(f) Results show the GluN2B/GluN2A ratio in APP immunoprecipitates in blots as shown in b) and are expressed as the mean ± SEM, relative to the adult group (One-way ANOVA, $p=0.049$, $F(2, 9)=4.296$, followed by Uncorrected Fisher's LSD's multiple comparisons test, *$p<0.05$, $n=4$).

(g) The effect of the presence of the APP C-terminal antibody in the intracellular space in neurons from infant mice (7-10 days) was evaluated using two concentrations of APP C-terminal antibody (Y188): 2.5 nM and 5 nM, introduced in the recording pipette. NMDAR EPSC amplitude was measured by whole-cell patch clamp in CA1 pyramidal neurons of infant C57BL/6 wild-type mice during 60 min of incubation with the antibody. The percentage of Y188-sensitive NMDAR EPSCs was calculated comparing the baseline amplitude (15-20min) with the final amplitude (60min) and normalized to the control condition (boiled Y188). Results are expressed as the mean ± SEM (Unpaired t-test, $n=7$-14).

(h) Time course of NMDAR EPSC amplitude measured by whole-cell patch clamp in CA1 pyramidal neurons of infant C57BL/6 wild-type mice with or without treatment with LY2811376, administrated 12h prior to patch-clamp recordings. NMDAR EPSCs were recording during 60 min of incubation with an antibody against the APP C-terminal (Y188), Results are expressed as the mean ± SEM (n=3-11).

The full statistical analysis and Western Blot membranes are provided in the Supporting Information.

Fig. S3

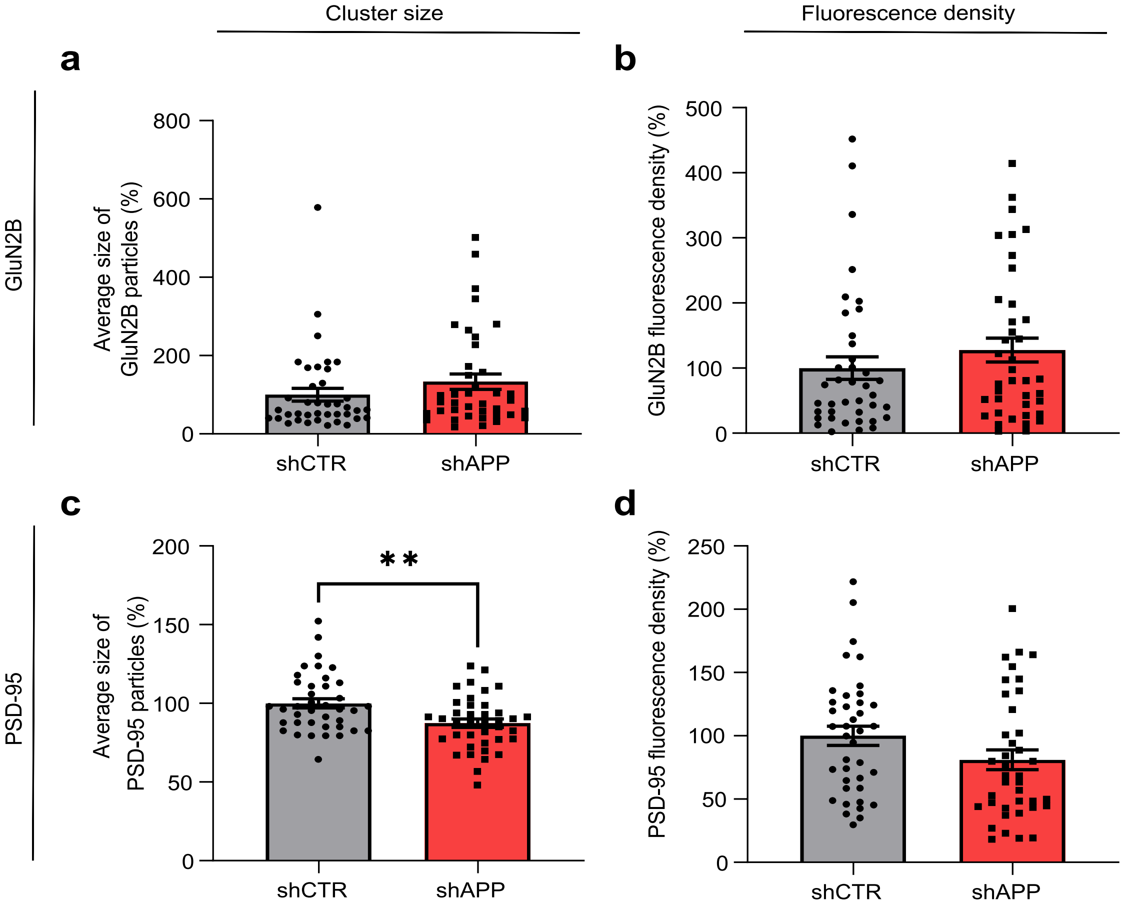
Cluster size
Fluorescence density
a
b
c
d
GluN2B
PSD-95
Average size of GluN2B particles (%)
GluN2B fluorescence density (%)
Average size of PSD-95 particles (%)
PSD-95 fluorescence density (%)
shCTR
shAPP
**

Supplementary Figure 3 – APP effects on PSD-95, GluN2B cluster size and GluN2B/PSD-95 total levels.

(a, b) Quantification of GluN2B average cluster size and fluorescence density in primary neuronal cultures (DIV14) transfected with shAPP or the respective control (shCTR) at DIV7, as shown in Figure 3c). Results are expressed as the mean ± SEM, using the control condition as reference (%) (Mann-Whitney test, n=39 dendrites, 3 independent cultures).

(c, d) Quantification of PSD-95 average cluster size and fluorescence density in primary neuronal cultures transfected with shAPP or the respective control (shCTR) as shown in Figure 3c). Results are expressed as the mean ± SEM, using the control condition as reference (%). c) Unpaired t-test, $^{**}p<0.01$. n=39 dendrites, 3 independent cultures; d) Mann-Whitney test, n=39 dendrites, 3 independent cultures.

The full statistical analysis is provided in the Supporting Information.

Fig. S4

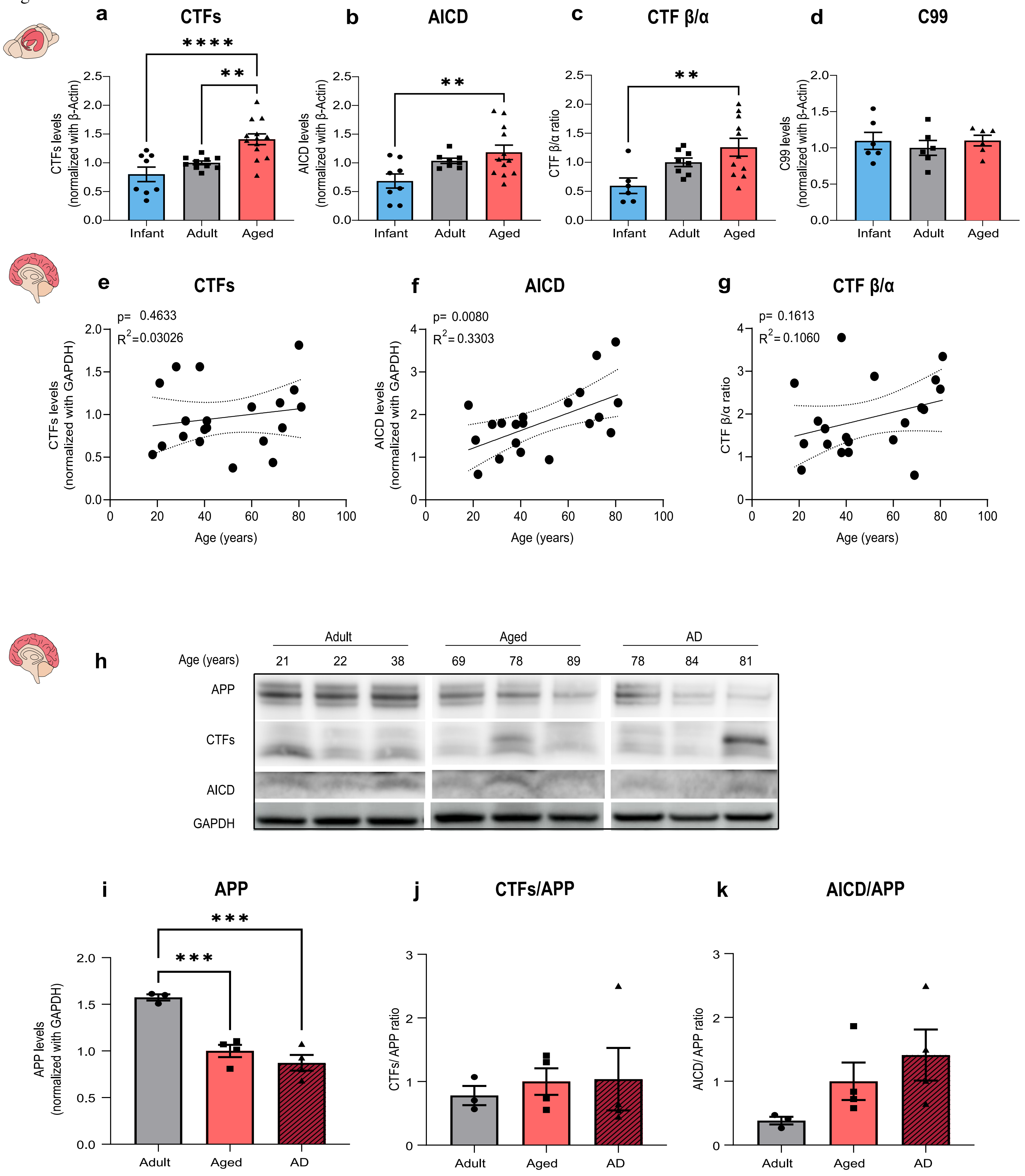
a
CTFs
CTFs levels
(normalized with β-Actin)
****
**
Infant
Adult
Aged
b
AICD
AICD levels
(normalized with β-Actin)
**
Infant
Adult
Aged
c
CTF β/α
CTF β/α ratio
**
Infant
Adult
Aged
d
C99
C99 levels
(normalized with β-Actin)
Infant
Adult
Aged
e
CTFs
p= 0.4633
R2=0.03026
CTFs levels
(normalized with GAPDH)
Age (years)
f
AICD
p= 0.0080
R2= 0.3303
AICD levels
(normalized with GAPDH)
Age (years)
g
CTF β/α
p= 0.1613
R2= 0.1060
CTF β/α ratio
Age (years)
h
Adult
Aged
AD
Age (years)
21
22
38
69
78
89
78
84
81
APP
CTFs
AICD
GAPDH
i
APP
APP levels
(normalized with GAPDH)
***
***
Adult
Aged
AD
j
CTFs/APP
CTFs/APP ratio
Adult
Aged
AD
k
AICD/APP
AICD/APP ratio
Adult
Aged
AD

Supplementary Figure 4 – APP processing throughout aging in mouse and human brain.

(a) Results from blots shown in Figure 4a) in hippocampal lysates from infant (7-10 days), adult (10 – 16 weeks) and aged (18 – 20 months) C57BL/6 wild-type mice. The levels of CTFs (normalized with β-actin) are expressed as the mean ± SEM relative to the adult group (One-way ANOVA, p=0.0002, F(2, 28)=12.13, followed by Uncorrected Fisher's LSD's multiple comparisons test, **p<0.01, ****p<0.001, n=8-13).

(b) Results from blots shown in Figure 4a) from mouse hippocampal lysates show the levels of AICD (normalized with β-actin) expressed as the mean ± SEM relative to the adult group, (One-way ANOVA, p=0.0155, F(2, 25)=4.946, followed by Uncorrected Fisher's LSD's multiple comparisons test, **p<0.01, n=8-13).

(c) Results from blots shown in Figure 4a) from mouse hippocampal lysates show the ratio between CTFβ and CTFα and are expressed as the mean ± SEM relative to the adult group (One-way ANOVA, p=0.0122, F(2, 22)=5.417, followed by Uncorrected Fisher's LSD's multiple comparisons test, **p<0.01, n=6-11).

(d) Results from blots shown in Figure 4e) from mouse hippocampal lysates show the levels of C99 (normalized with β-actin) and are expressed as the mean ± SEM relative to the adult group (One-way ANOVA, p=0.7254, F(2, 15)=0.3281, n=8-13).

(e) Linear regression graph calculated from blots as shown in Figure 4g) shows the variation in the levels of APP CTFs (normalized with the loading control GAPDH) depending on the age of human subjects. Statistical analysis was performed using Pearson's correlation (two-tailed p value), p=0.4633, R^2=0.03026, n=20. Dotted lines represent the 95% confidence intervals. The values obtained for 20–25-year-old subjects were used as reference.

(f) Linear regression graph calculated from blots as shown in Figure 4g) shows the variation in the levels of AICD (normalized with the loading control GAPDH) depending on the age of human subjects. Statistical analysis was performed

using Pearson's correlation (two-tailed p value), p=0.0080, R^2=0.3303, n=20. Dotted lines represent the 95% confidence intervals. The values obtained for 20–25-year-old subjects were used as reference.

(g) Linear regression graph calculated from blots as shown in Figure 4g) shows the variation in the ratio between CTFβ and CTFα depending on the age of human subjects. Statistical analysis was performed using Pearson's correlation (two-tailed p value), p=0.1613, R^2=0.1060, n=20. Dotted lines represent the 95% confidence intervals. The values obtained for 20–25-year-old subjects were used as reference.

(h) Representative western blot of prefrontal cortex human samples (21 to 89 years old), with or without Alzheimer's Disease (AD). Membranes were immunoblotted with antibodies for APP C-terminal (to detect APP full-length (APP), APP C-terminal fragments (CTFs) and the APP Intracellular Domain (AICD)) and GAPDH as the loading control. Some of the samples in Figure 4g) served as internal controls among groups.

(i) Quantification of APP levels in human samples (normalized to GAPDH) calculated from blots as shown in h), comparing adult subjects (<40 years old), aged subjects (≥65 years old) and AD patients (≥65 years old). Results are expressed as the mean ± SEM, using the aged subjects as reference (One-way ANOVA, p=0.0003, $F(2, 8)$=25.33, followed by Uncorrected Fisher's LSD multiple comparisons test, ***p<0.001, n= 3-4).

(j) Quantification of CTFs/APP levels in human samples calculated from blots as shown in h), comparing adult subjects (<40 years old), aged subjects (≥65 years old) and AD patients (≥65 years old). Results are expressed as the mean ± SEM, using the aged subjects as reference (Kruskal Wallis p=0.6038, Kruskal-Wallis statistic=1.136, n=3-4).

(k) Quantification of AICD/APP levels in human samples calculated from blots as shown in h), comparing adult subjects (<40 years old), aged subjects (≥65 years old) and AD patients (≥65 years old). Results are expressed as the mean

± SEM, using the aged subjects as reference (One-way ANOVA $p=0.1489$, $F(2, 8)= 2.439$, $n=3-4$).

The full statistical analysis and Western Blot membranes are provided in the Supporting Information.

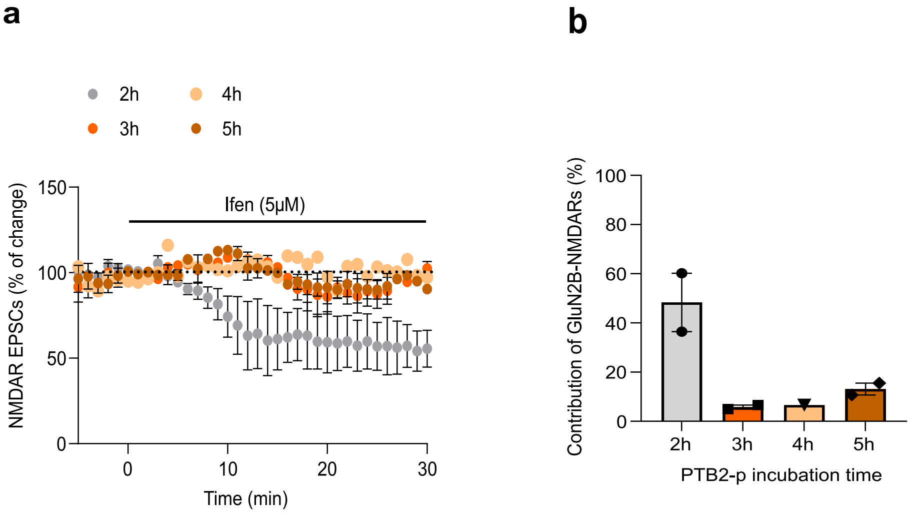
a
2h
4h
3h
5h
Ifen (5μM)
NMDAR EPSCs (% of change)
150
100
50
0
0
10
20
30
Time (min)
b
Contribution of GluN2B-NMDARs (%)
100
80
60
40
20
0
2h
3h
4h
5h
PTB2-p incubation time

Supplementary Figure 5 –Time course of PTB2-p incubation.

(a) Time course of ifenprodil (5μM) effect on pharmacologically isolated NMDAR EPSC amplitude in CA1 pyramidal neurons, measured by whole-cell patch clamp in aged C57BL/6 wild-type mice (PTB2-p 5nM incubation for 2, 3, 4 or 5h prior to patch-clamp recordings, n=1-2).

(b) GluN2B contribution was calculated as the percentage of change in NMDAR EPSCs after ifenprodil perfusion (for 30 min) in aged C57BL/6 wild-type mice (PTB2-p incubation (5nM) for 2, 3, 4 or 5h prior to patch-clamp recordings, n=1-2).

1) Detailed description of Materials and Methods

Animals

Animal procedures were performed at the Rodent Facility of Instituto de Medicina Molecular, that is a licensed establishment (license number 017918/2021) in compliance with the European Directive 2010/63/EU, transposed to Portuguese legislation in DL 133/2013. All animal research projects carried out at iMM are reviewed by the Animal Welfare Body (ORBEA-iMM) to ensure that the use of animals is carried out in accordance with the applicable legislation and following the 3R's principle. Environmental conditions were kept constant: food and water ad libitum, 22-24 ºC, 45-65% relative humidity, 14h light/10 dark cycles, 3 to 4 mice per cage.

Experiments performed at IPMC were done according to policies on the care and use of laboratory animals of European Communities Council Directive (2010/63) and the protocols were approved by the French Research Ministry following evaluation by a specialized ethics committee (protocol number 00973.02) All efforts were made to minimize animal suffering and reduce the number of animals used. The animals were housed three per cage under controlled laboratory conditions with food and water ad libitum, a 12 hr dark light cycle and a temperature of 22 ±2°C.

Patch Clamp electrophysiology

Newborn mice were anesthetised through hypothermia, whereas adult and aged mice were anesthetized [ketamine (150 mg/kg)/xylazine (10 mg/kg)]. All groups were transcardially perfused with artificial cerebrospinal fluid (aCSF) for slice preparation. Acute transverse hippocampal slices (250 µm) from wild-type C57BL/6 mice were prepared on a vibratome (Microm HM600V, Thermo Scientific, France) in ice-cold dissecting solution containing (in mM): 234 sucrose, 2.5 KCl, 0.5 CaCl2, 10 $MgCl_2$, 26 NaHCO3, 1.25 NaH_2PO_4 and 11 D-glucose, oxygenated with 95% O_2 and 5% CO_2, pH 7.4. Slices were incubated for 60 min at 37°C, in an artificial CSF (aCSF) solution containing (in mM): 119 NaCl, 2.5

KCl, 1.25 NaH_2PO_4, 26 $NaHCO_3$, 1.3 $MgSO_4$, 2.5 $CaCl_2$ and 11 D-glucose, oxygenated with 95% O2 and 5% CO2, pH 7.4. Slices were used after recovering for another 30 min at room temperature. To measure pharmacologically isolated NMDAR EPSCs, slices were perfused with the oxygenated aCSF at 31 ± 1°C in the continuous presence of 50 mM picrotoxin (Sigma-Aldrich, dissolved in DMSO) to block GABAergic transmission and DNQX (10 µM) to block AMPA receptors.

Recording pipettes (5-6 MΩ) for voltage-clamp experiments were filled with a solution containing the following: 117.5 mM Cs-gluconate, 15.5 mM CsCl, 10 mM TEACl, 8 mM NaCl, 10 HEPES, 0.25 mM EGTA, 4 mM MgATP and 0.3 NaGTP (pH 7.3; osmolarity 290-300 mOsm). Slices were visualized on an upright microscope with IR-DIC illumination (Scientifica, Ltd). Whole-cell recordings were performed using a Multiclamp 700B (Molecular Devices) amplifier, under the control of pClamp10 software (RRID:SCR_011323) (Molecular Devices). The Schaffer collateral pathway was stimulated at 0.10 Hz using electrodes (glass pipettes filled with aCSF) placed in the stratum radiatum.

After a tight seal (>1 GW) on the cell body of the selected neuron was obtained, whole-cell patch clamp configuration was established, and cells were left to stabilize for 2–3 min before recordings began. Pharmacologically isolated NMDAR EPSCs were recorded from cells voltage clamped at +40 mV. Holding current and series resistance were continuously monitored throughout the experiment, and if either of these two parameters varied by more than 20%, the experiment was discarded. Electrical stimulation was adjusted to elicit EPSCs of approximately 150 pA amplitude in the different studied groups.

Fractionation into PSD-enriched fractions

Hippocampi from C57BL/6 wild-type mice were dissected and snap-frozen in liquid nitrogen. All centrifugation steps were performed at 4°C and all solutions contained protease/phosphatase inhibitors. Samples were first homogenized using a Glass/Teflon Potter Elvehjem homogenizer in Buffer I (0.32M sucrose and 10mM HEPES, pH 7.4) and then centrifugated (1000 g for 10min) to remove nuclei and cell debris. This centrifugation step was repeated 3 times, until the supernatant was completely clear and finally subjected to a centrifugation at

12.000 g for 20min. The pellet was recovered, resuspended in Buffer II (4mM HEPES and 1 mM EDTA, pH 7.4) and centrifuged twice at 12000 g for 20 min. The pellet was then resuspended in 25µL of Buffer III (20 mM HEPES, 100mM NaCl, 0.5% Triton X100, pH= 7.2) and incubated 1h at 4°C with mild agitation. By centrifuging the samples at 12000 g for 20 min it was possible to pellet the synaptosome membrane fraction, whereas the supernatant was collected as the non-postsynaptic density membrane fraction (non-PSD) or Triton-soluble fraction. Finally, the pellet was solubilized in 25µL Buffer IV (20 mM HEPES, 0.15 mM NaCl, 1% TritonX-100, 1% sodium deoxycholate, 1% SDS, pH 7.5) for 1 h at 4°C and centrifuged at 10000 g for 15 min. The supernatant contained the PSD or Triton-insoluble fraction. When analyzing PSD-enriched fractions, the levels of the proteins of interest (GluN2B, GluN2A and APP) were normalized to PSD-95 to account for the possible variability in protein loading, PSD fractionation efficiency and number of synapses at each age. With this type of normalization, our output corresponds to the protein levels per PSD-95, therefore reflecting the synaptic composition rather than the absolute values.

Synaptosomes preparation

All centrifugation steps were performed at 4°C and all solutions contained protease/phosphatase inhibitors. Hippocampi from C57BL/6 wild-type mice were dissected, resuspended in a 0.32 M sucrose solution containing 50mM Tris, 2 mM EDTA, pH=7.6 and homogenized using a Glass/Teflon Potter Elvehjem homogenizer. The suspension was centrifuged at 3000 g during 10 min. The supernatant was collected and centrifuged at 1000 g for 12 min. The pellet was resuspended in 1.8 ml of a 45% vol/vol Percoll solution made up in a Krebs-Ringer solution (140mM NaCl, 1mM EDTA, 10mM HEPES, 5mM KCl, pH= 7.4). After centrifugation at 21100g for 2 min, the top layer was collected (synaptosome fraction) and washed twice in Krebs-Ringer solution (centrifugation at 21100g, 2 min). When synaptosome fractions were used for co-immunoprecipitation experiments, they were subsequently resuspended in the respective buffer (50mM Tris HCl pH 7.5; 150 mM NaCl; 2 mM EDTA; 1% Triton with protease and phosphatase inhibitors).

Extraction of soluble and membrane/cytosolic proteins

Hippocampal mouse tissue was resuspended in DEA buffer (50mM NaCl, 0.2% Diethylamine, pH 10, with protease and phosphatase inhibitors) and homogenised using Precellys (6500rpm for 30s). Samples were then centrifuged for 10min at 5000g at 4°C to pellet membrane, nuclei and mitochondria (pellet 1), which was resuspended in RIPA buffer (see below). The supernatant was collected and ultra-centrifuged for 30min at 130000g at 4°C. The resulting supernatant (DEA fraction, containing soluble proteins) was collected, the pH was adjusted by adding 10% of 0.5M Tris, pH 6.8 and samples were stored for analysis. The pellet (pellet 2) was further dissolved in RIPA buffer and used in the next steps of the protocol (see below).

The pellet 1 was resuspended in RIPA buffer (20mM Tris-HCl, pH 7.5, 150mM NaCl, 1mM EDTA, 1mM EGTA, 1% NP-40, 1% sodium deoxycholate, 2.5mM sodium pyrophosphate (with protease and phosphatase inhibitors) and homogenised using Precellys (5000rpm for 30s). Samples were then centrifuged for 10min at 5000g at 4°C to remove insoluble material. The resulting supernatant was combined with the pellet 2 of the previous ultra-centrifugation. Samples were then ultracentrifuged for 60min at 130000g at 4°C. Finally, the supernatant, which corresponds to the RIPA fraction, containing membrane/cytosolic proteins, was collected and used for further analysis.

Co-immunoprecipitation

Frozen tissue was resuspended in immunoprecipitation buffer (50mM Tris HCl pH 7.5; 150mM NaCl; 2mM EDTA; 1% Triton with protease and phosphatase inhibitors) and homogenized using a Glass/Teflon Potter Elvehjem homogenizer. Following a centrifugation at 1000g, 10 min 4°C the supernatant was collected. Protein quantification of total lysates and synaptosome fractions was performed using the BioRad DC Protein assay kit. The immunoprecipitation protocol was adapted from (Tomé et al., 2021). For each sample, 50µL of Dynabeads were washed 3 times with washing buffer (0.1% BSA; 2mM EDTA in PBS). Dynabeads were then resuspended in 500uL washing buffer and the appropriate volume of antibody: APP C-terminal Y188 (3µg, ab32136, Abcam), PSD95 (3µg, ab18258,

Abcam) or Normal rabbit IgG (3µg, 12-370, Merck Millipore) and incubated overnight at 4°C under rotation. Following 3 washing steps with washing buffer, dynabeads were resuspended in 350uL washing buffer and incubated with 500µg of protein lysate diluted in Immunoprecipitation buffer (500µL) for 2h at 4°C under rotation. Following 5 washing steps with Immunoprecipitation buffer, dynabeads were gently resuspended in 60µL of pre-heated 2x sample buffer (140mM Tris HCl pH 6.8, 4% SDS, 13.6% glycerol, 272mM DTT, 0.004% Blue bromophenol) in RIPA (50mM Tris, 1mM EDTA, 150mM NaCl, 0.1% SDS, 1%Tergitol-type NP-40, pH 8.0). Finally, samples were incubated for 10 min at 95°C, the supernatant was collected and used for Western Blot analysis.

Western blotting

Mouse and human frozen tissue samples were resuspended in A-EDTA buffer (10 mM HEPES-KOH pH 7.9, 10 mM KCl, 1.5 mM $MgCl_2$, 0.1 mM EDTA, 0.3% NP-40 with protease and phosphatase inhibitors) and homogenized using a Glass/Teflon Potter Elvehjem homogenizer, as described in (Pousinha et al., 2017). Following protein quantification using BioRad DC Protein assay kit, lysates were diluted in water and 5x sample buffer (Final concentration: 50mM Tris HCl pH 6.8, 2% SDS, 6% glycerol, 0.1% bromophenol blue, 121mM DTT) and denatured at 95°C for 10 min.

For APP, APP-CTFs and AICD analysis, proteins were separated using precast gradient Tricine Protein Gels (10–20%, 1 mm, Novex) in Tris-tricine buffer (1M Tris, 1M Tricine, 1% SDS). Samples were electro-transferred at 400mA for 1h to 0.2µm Nitrocellulose membranes using a Tris Glycine buffer (25 mM Tris, 190 mM glycine) with 20% ethanol. Proteins transferred to nitrocellulose membranes were additionally denatured by boiling the membrane in PBS for 5 min, acting as an antigen retrieval step to detect AICD, as described in (Pimplikar & Suryanarayana, 2011).

For all the remaining proteins, electrophoresis was performed in Tris-glycine buffer with 10% SDS using 10-12% and 4% acrylamide resolving and stacking gels, respectively. Proteins were electro-transferred to 0.45µm Polyvinylidene

fluoride (PVDF) membranes in Tris-glycine buffer with 20% methanol at 350mA for 90 min.

After transfer, all membranes were blocked with 3% BSA in TBS-T (20 mM Tris,150 mM NaCl, 0.1% Tween-20) at room temperature (RT) for 1h and incubated with primary antibodies (diluted in 3% BSA TBS-T) overnight at 4°C. The following antibodies were used: APP C-terminal Y188 (1:1000, ab32136, Abcam), APP M3.2 (1:1000, 805702, Biolegend), Phospho-GluN2B (1:1000, #4208, Cell Signalling), GluN2A (1:200, sc-136004, Santa Cruz), GluN2B (1:1000, D15B3, Cell Signalling), PSD-95 (1:1000, D27E11, Cell signalling), Synaptophysin (1:200, S5768, Merck Millipore), β-actin (1 :1000, sc-47778, Santa Cruz), GAPDH (1:1000, AM4300, Invitrogen). After three washing steps of 10min with TBS-T, membranes were incubated with horseradish peroxidase (HRP)—conjugated anti-mouse or anti-rabbit secondary antibodies for 1 h at RT: Goat Anti-Mouse IgG HRP (1:4000, 10004302, Cayman Chemicals) or Goat Anti-Rabbit IgG HRP (1:10000, 1706515, Bio-Rad). After three washing steps of 10min with TBS-T, chemiluminescent detection was performed with enhanced chemiluminescence (ECL) western blotting detection reagent (GE Healthcare). For AICD detection, longer exposure times were applied.
For the analysis of soluble APP fragments (sAPPβ and α), DEA fractions were loaded in the gel, followed by electrophoresis in Tris-glycine buffer with 10% SDS. Following transfer, membranes were blocked in I-Block solution (1g Topix I-Block, Thermo Fischer Scientific, in 500ml PBS, 0.2% Tween20) for 1h at RT and incubated with the following primary antibodies diluted in I-Block solution overnight at 4°C: sAPPβ (1:2000, 18957, IBL), sAPPα (1:100, 18058, Biolegend), β-Actin (1:5000, A5316, Sigma). The washing steps were performed with PBS-Tween buffer and the secondary antibodies were diluted in I-Block solution and incubated for 1h at RT. For ECL detection, membranes were incubated for 1 min at RT with peroxidase substrate (Western lightning ultra, PerkinElmer) and signals were captured with phospho-Fusion imager, Vilber Lourmat.

Optical density was determined with Image-J, according to the software instructions (T. Ferreira. & Rasband, 2012).

Primary neuronal cultures

18-day Sprague-Dawley rat embryos were collected in Hank's Balanced Salt Solution (HBSS, Corning) and rapidly decapitated. After removing the meninges, hippocampi were dissociated in HBSS with 0.25% trypsin at 37°C for 15 min, resuspending every 3min. The tissue was then washed with HBSS containing 30% fetal bovine serum (FBS) to stop trypsin activity, followed by three washing steps with HBSS. Cells were resuspended in neuronal plating medium (MEM (Minimum Essential Medium) supplemented with 10% horse serum, 0.6% glucose, and 100 U/mL Pen-Strep), gently dissociated and filtered through a 70μm strainer. Finally, cells were plated on poly-D-lysine-coated glass coverslips (0.1mg/mL PDL in 0.1M borate buffer, pH 8.5) in 24-multi well plates at a final density of 70000 cells/coverslip, in neuronal plating media and maintained at 37°C in a 5% CO_2-humidified incubator. After 4 hours, the plating medium was replaced for neuronal culture medium: Neurobasal Medium (Gibco–Life Technologies) supplemented with B-27 supplement, 25μM Glutamic acid, 0.5mM Glutamine, and 20 U/ml penicillin/streptomycin. Cultures were maintained in the humidified incubator for 2 weeks, feeding the cells once per week with neuronal culture medium by replacing half of the medium per well.

Neuronal transfection

Primary neuronal cultures were transiently transfected at DIV (days *in vitro*) 7-8 using the calcium phosphate transfection protocol. For each well, 1.5μg plasmid DNA was diluted in 17.5μL TE buffer (10 mM Tris, 1 mM EDTA, pH 7.3). $CaCl_2$ solution (2.5M in 10mM HEPES, pH 7.2) was added dropwise to the diluted DNA (final concentration = 250 mM $CaCl_2$) and gently mixed. This mix was then added dropwise to an equivalent volume of HEPES-buffered saline transfection solution (in mM: 274 NaCl, 10 KCl, 1.4 Na_2HPO_4, 11 dextrose, 42 HEPES, pH 7.2), gently mixed and incubated at room temperature for 30 min, vortexing every 5 min. During this period, neurons were treated with 2 mM kynurenic acid in conditioned neuronal culture medium (in a new multi-well plate). The precipitates were then added dropwise to pre-conditioned neurons, followed by an incubation of 2-3h at

37°C in a 5% CO_2-humidified incubator. Finally, DNA precipitates were dissolved by incubating the neurons in an acidified neuronal culture medium (in mM: 2 kynurenic acid, ~5 HCl final concentration) for 15-20 min at 37ºC. Coverslips were transferred to the original plates with conditioned neuronal culture media maintained in the humidified incubator.

Generation of the shRNA-APP construct

Briefly, the shRNA insert was generated by PCR amplification using primers with AscI and XhoI restriction sites (Forward Primer: AscI-U6 5'-GCGGCGCGCCAGGAAGAGGGCCTATTTCCCATG-3'; Reverse Primer: XhoI-PolyA-Active 5'-GCAAGTTAGTGCTTTTTCTAGACCCTCGAGCG-3'). Subsequently, the PCR product and the AAV-U6-shRNAempty-CMV-mCherry plasmid were digested with AscI and XhoI restriction enzymes. Following gel purification, the shRNA construct was ligated into the AAV plasmid and the ligation product was transformed into Top10 chemically competent cells.

Immunocytochemistry

Neurons were fixed at DIV 14-15 (7 days after transfection) in 4% sucrose and 4% paraformaldehyde in phosphate-buffered saline (PBS) for 20 min at room temperature (RT). Neurons were then washed 3 times with PBS and permeabilized with PBS + 0.25% (v/v) Triton X-100 at RT (for 10min in the case of intracellular epitopes and 5 min for extracellular targets). Following 3 washing steps in PBS, cells were incubated in 10% (w/v) bovine serum albumin (BSA) in PBS for 1 h at RT to block nonspecific staining. Incubation with primary antibodies was performed overnight at 4°C in a humidified chamber, with antibodies diluted in 3% BSA PBS: APP Y188 (1:100, ab32136, Abcam), GluN2B (1:100, AGC-003, Alomone), GluN2A (1:100, AGC-002, Alomone), PSD-95 (1:50, ADI-VAM-PS002-E, Enzo). Following 4 washing steps in PBS, cells were incubated with the appropriate secondary antibody diluted in 3% BSA PBS (1:500) for 1h at RT: Donkey anti-Rabbit IgG Alexa Fluor 488, Donkey anti-Mouse IgG Alexa Fluor 488 or Donkey anti-Rabbit Alexa Fluor 647 (Thermo Fisher). Finally, cells were stained with Hoechst 33258 (12 ug/mL in PBS, Life Technologies) for 5min,

washed 3 times with PBS and mounted in Fluoromount aqueous mounting medium.

Microscopy imaging and analysis

All images were acquired in a Zeiss LSM 880 laser scanning confocal microscope using a Plan-Apochromat 63x/1.4 oil immersion objective.

For the analysis of APP immunofluorescence in transfected neurons, Hoechst fluorescence was detected using 405 nm for excitation (Diode laser with 30 mW nominal output – 2% transmission) and a 415-475 nm detection window, with PMT gain set to 610 and offset to -1. Alexa Fluor 488 fluorescence was detected using the 488 nm laser line of an Ar laser for excitation (25 mW nominal output – 1% transmission) and a 498-557 nm detection window, with GaAsP detector gain set to 500 and offset to 1. mCherry fluorescence was detected using 594 nm for excitation (HeNe laser with 2 mW nominal output – 5% transmission) and a 600-735 nm detection window, with PMT gain set to 700 and offset to 1. The pinhole size was set to 1.67 AU for Hoechst, 1.37 AU for Alexa Fluor 488 and 1.1 AU for mCherry. Z-stacks of the three channels were acquired with Zoom set to 1 (134.95x134.95 μm area with 1024x1024 pixel frame size - 0.13 μm pixel size) with a 0.49 μm slice interval, a line average of 2 and 1.03 μs pixel dwell time (unidirectional scan). The APP (Alexa Fluor 488) relative fluorescence intensity was manually quantified using ImageJ, after maximum intensity projection. For each condition, 7 transfected neurons were analyzed by defining regions of interest (ROI) which corresponded to the cell bodies using the mCherry channel. The average intensity of Alexa Fluor 488 was then determined for each ROI. All values were normalized to the average intensity in transfected neurons from the control condition (%).

For the analysis of GluN2B/PSD95 in dendrites of transfected neurons, Alexa Fluor 488 fluorescence was detected using the 488 nm laser line of an Ar laser for excitation (25 mW nominal output – 3% transmission) and a BP 495-550 + LP 570 nm filter set for detection in the Airyscan unit, with gain set to 790 and offset to 0. mCherry fluorescence was detected using 561 nm for excitation (DPSS laser with 20 mW nominal output – 7.5% transmission) and a BP 495-550 + LP 570 nm

filter set in conjunction with a SP 615 nm secondary beam splitter for detection, with gain set to 850 and offset to 0 in the Airyscan unit. Alexa Fluor 647 fluorescence was detected using 633 nm for excitation (HeNe laser with 5 mW nominal output – 14.5% transmission) and a BP 570-620 + LP 645 nm filter set in conjunction with a LP 660 nm secondary beam splitter for detection with gain set to 760 and offset to 0 in the Airyscan unit. The pinhole size was set to 2.66 AU for Alexa Fluor 647, 2.16 AU for mCherry and 2.49 AU for Alexa Fluor 488. Z-stacks of the three channels were acquired with Zoom set to 3.8 (35.51x35.51 µm area with 836x836 pixel frame size - 0.04 µm pixel size) with a 0.18 µm slice interval, a line average of 2 and 2.53 µs pixel dwell time (unidirectional scan). All data sets were subjected to Airyscan processing in ZEN using the same parameters. The analysis of GluN2B/PSD95 in transfected neurons was performed using an in-house developed macro for ImageJ. For each condition, 13 dendrites from transfected neurons were analyzed. Following maximum intensity projection and manual selection of the dendritic area using the mCherry channel, images were segmented with user-defined intensity thresholds for GluN2B (Alexa Fluor 647) and PSD95 (Alexa Fluor 488), which were maintained constant for all conditions. The mean fluorescence intensity and the percentage of dendritic area with positive signal (above threshold) were quantified for each channel in the segmented images. GluN2B and PSD95 clusters were detected by particle analysis, which also allowed us to quantify average cluster sizes in each case. The colocalization area was determined by identifying the pixels where both GluN2B and PSD95 intensity values were above the respective threshold. The relative GluN2B synaptic content was then determined as the ratio between the area occupied by colocalized pixels and the total area with GluN2B staining. The fluorescence density was calculated as the mean fluorescence intensity multiplied by the percentage of dendritic area with positive signal for each channel. All quantifications were normalized to the average values of transfected dendrites from the control condition (%).

Supplementary Table 1 - Information on human samples

Age	Gender	Post mortem delay	Neuropathological diagnosis
18	F	81h	-
21	M	≥30h	-
22	M	≥30h	-
28	M	12h	-
31	M	36h	-
32	M	48h	-
38	M	24h	-
38	M	24h	-
40	F	36h	-
41	M	24h	-
41	M	24h	-
52	F	29h	-
60	M	≥30h	-
65	M	≥30h	-
69	M	6h	-
72	M	29h	-
73	M	22h	-
78	M	23h	-
80	F	25h	-
81	F	75h	-
89	F	12h	-
65	M	46h	AD (Braak stage VI)
78	M	18h	AD (Braak stage VI)
84	M	Not determined	AD (Braak stage VI)
81	F	41h	AD (Braak stage VI)

Supplementary Table 2 - Complete statistical analysis

Figure 1 - GluN2B-NMDAR synaptic contribution is increased in infant and aged mice

Figure	Experiment	Condition	number of animals	number of cells	Measurement	Treatment	Mean	SEM	Units	Normality (Shapiro Wilk test)	Statistical test	p value	F/t/z/R ETC value	pos hoc Test	Comparison	p value	
**1c**	τweighted	Infant	_	44	τw = [(af.τf) + (as.τs)]/(af + as)	_	156,200	4,201	ms	yes	Kruskal-Wallis test	0,0040	Kruskal-Wallis statistic= 11,02	Uncorrected Dunn's test	Adult vs. Infant	0,0052	**
		Adult		28			126,300	9,111		yes					Adult vs. Aged	0,0021	**
		Aged		33			177,000	12,660		no					Infant vs. Aged	0,6185	
**1f**	Contribution of GluN2B to NMDAR EPSCs	Infant	_	12	Amplitude (5 minutes before and 25-20 min after ifenprodil perfusion)	Ifenprodil (5µM)	50,880	5,581	pA (%)	yes	Ordinary One-way ANOVA	0,0002	F (2, 28) = 11,37	Uncorrected Fisher's LSD	Adult vs. Infant	<0,0001	****
		Adult		10			14,200	4,322		yes					Adult vs. Aged	0,0298	*
		Aged		9			33,110	6,727		yes					Infant vs. Aged	0,0331	*
**1i**	GluN2A relative (mice, PSD fractions)	Infant	5	_	Optical density (GluN2A normalized with PSD-95)	_	0,797	0,226	A.U.	yes	Ordinary One-way ANOVA	0,5735	F (2, 12) = 0,5825	_	_	_	_
		Adult	5				1,000	0,069		yes							
		Aged	5				0,992	0,110		yes							
**1j**	GluN2B relative levels (mice, PSD fractions)	Infant	5	_	Optical density (GluN2B normalized with PSD-95)	_	2,974	0,783	A.U.	yes	Ordinary One-way ANOVA	0,0140	F (2, 12) = 6,224	Uncorrected Fisher's LSD	Adult vs. Infant	0,0096	**
		Adult	5				1,000	0,046		yes					Adult vs. Aged	0,9635	
		Aged	5				1,030	0,044		yes					Infant vs. Aged	0,0104	*
**1k**	GluN2B/GluN2A relative ratio (mice, PSD fractions)	Infant	5	_	Optical density (GluN2B/GluN2A)	_	4,300	0,938	A.U.	yes	Kruskal-Wallis test	0,0024	Kruskal-Wallis statistic=9,42	Uncorrected Dunn's test	Adult vs. Infant	0,0109	*
		Adult	5				1,000	0,037		yes					Adult vs. Aged	0,832	
		Aged	5				1,081	0,131		no					Infant vs. Aged	0,0058	**

Figure 2 - APP interacts and regulates GluN2B-NMDARs at immature synapses

Figure	Experiment	Condition	number of animals	number of cells	Measurement	Treatment	Mean	SEM	Units	Normality (Shapiro Wilk test)	Statistical test	p value	F/t/z/R ETC value	pos hoc Test	Comparison	p value	
**2b**	APP relative levels (mice, PSD fractions)	Infant	8	_	Optical density (APP normalized with PSD-95)	_	5,521	0,8299	A.U.	no	Kruskal-Wallis test	0,0005	Kruskal-Wallis statistic = 15,37	Uncorrected Dunn's test	Adult vs. Infant	0,0006	***
		Adult	8				1	0,06573		yes					Adult vs. Aged	0,9436	
		Aged	8				1,014	0,07415		yes					Infant vs. Aged	0,0008	***
**2g**	Reduction in NMDAR EPSCs after APP blocking (%)	Infant	_	15	Amplitude (55-60 min comparing to baseline (15-20min)	APPCt, clone Y188 (2.5nM, 0-60min) / control	22,36	4,346	pA (%)	yes	Ordinary One-way ANOVA	<0,0001	F (3, 40) = 12,65	Uncorrected Fisher's LSD	Infant vs. Infant LY2811376	0,1378	***
		Infant LY2811376		11			31,97	5,801		yes					Adult vs. Infant	0,0003	***
		Adult		8			-5,388	4,545		yes					Adult vs. Aged	0,526	
		Aged		10			-0,5357	3,95		yes					Infant vs. Aged	0,0011	**
**2j**	Reduction in NMDAR EPSCs after APP blocking + ifenprodil (%)	No antibody	_	6	Amplitude (85-90 min comparing to baseline (55-60min)	APPCt, clone Y188 (2.5nM, 0-90min)/ control + Ifenprodil (5μM, 60-90min)	53,28	6,984	pA (%)	yes	Ordinary One-way ANOVA	0,0019	F (2, 13) = 10,59	Uncorrected Fisher's LSD	No antibody vs. Boiled Y188	0,441	
		Boiled Y188		4			46,82	5,16		yes					No antibody vs. Y188	0,0007	***
		Y188		6			21,21	3,055		yes					Boiled Y188 vs. Y188	0,0077	**

Figure 3 - APP modulates the GluN2B-NMDAR synaptic content in immature neurons

Figure	Experiment	Condition	number of cells/dendrites	number of independent neuronal cultures	Measurement	Mean	SEM	Units	Normality (Shapiro Wilk test)	Statistical test	p value	F/t/z/R ETC value	
**3b**	APP relative immunoreactivity (%)	shCTR	20	3	APP immunofluorescence	100	8,155	A.U. (%)	yes	Mann Whitney test	<0,0001	Mann-Whitney U = 0	****
		shAPP	21	3		18,21	1,059		no				
**3d**	Relative GluN2B synaptic content (%)	shCTR	39	3	Co-localization area / GluN2B area	100	6,944	μm^2 (%)	no	Mann Whitney test	0,0209	Mann-Whitney U = 530	*
		shAPP	39	3		79,36	6,742		no				
**3e**	Relative GluN2B positive area (%)	shCTR	39	3	GluN2B total area	100	14,03	μm^2 (%)	no	Mann Whitney test	0,2777	Mann-Whitney U = 651	
		shAPP	39	3		120,9	14,27		no				
**3f**	Relative PSD-95 positive area (%)	shCTR	39	3	PSD-95 total area	100	6,767	μm^2 (%)	yes	Unpaired t test	0,0462	t=2,027, df=76	*
		shAPP	39	3		80,89	6,563		yes				

Figure 4 - Age is associated with an increase in APP processing in mice and humans

Figure	Experiment	Condition	number of mice/ human subjects	Measurement	Mean	SEM	Units	Normality (Shapiro Wilk test)	Statistical test	p value	F/t/z/R ETC value	pos hoc Test	Comparison	p value	
**4b**	APP relative levels (mice, whole lysates)	Infant	8	Optical density (APP normalized with β-actin)	0,9812	0,03091	A.U.	yes	Ordinary One-way ANOVA	0,8263	F (2, 28) = 0,1921	_	_	_	_
		Adult	10		1	0,017		yes							
		Aged	13		0,9755	0,0339		yes							
**4c**	C-terminal fragments/APP relative ratio (mice, whole lysates)	Infant	8	Optical density (CTFs/APP)	0,7947	0,1104	A.U.	yes	Kruskal-Wallis test	0,0008	Kruskal-Wallis statistic = 14,21	Uncorrected Dunn's test	Adult vs. Infant	0,333	
		Adult	10		1	0,03761		no					Adult vs. Aged	0,0077	**
		Aged	13		1,455	0,09908		yes					Infant vs. Aged	0,0004	***
**4d**	AICD/APP relative ratio (mice, whole lysates)	Infant	8	Optical density (AICD/APP)	0,6624	0,1019	A.U.	yes	Ordinary One-way ANOVA	0,0143	F (2, 25) = 5,057	Uncorrected Fisher's LSD	Adult vs. Infant	0,0511	
		Adult	8		1,042	0,05003		yes					Adult vs. Aged	0,3702	
		Aged	12		1,197	0,1435		yes					Infant vs. Aged	0,0041	*
**4f**	C99/APP relative ratio (mice, whole lysates)	Infant	6	Optical density (C99/APP)	0,783	0,1167	A.U.	yes	Ordinary One-way ANOVA	0,0802	F (2, 15) = 3,000	_	_	_	_
		Adult	6		1	0,07099		yes							
		Aged	6		1,121	0,1036		yes							
**4h**	Correlation between APP relative levels and age (humans, whole lysates)	_	20	Optical density (APP normalized with GAPDH)	_	_	A.U.	_	Pearson Correlation	0,0456	R^2= 0,204	_	_	_	_
**4i**	Correlation between C-terminal fragments/APP relative ratio and age (humans, whole lysates)	_	20	Optical density (CTFs/APP)	_	_	A.U.	_	Pearson Correlation	0,0192	R^2 = 0,2688	_	_	_	_
**4j**	Correlation between AICD/APP relative ratio and age (humans, whole lysates)	-	20	Optical density (AICD/APP)	_	_	A.U.	_	Pearson Correlation	0,0028	R^2 = 0,3986	_	_	_	_

Figure 5 - Age-related increase in βAPP processing contributes to enhanced GluN2B synaptic contribution

Figure	Experiment	Condition	number of cells	Measurement	Treatment	Mean	SEM	Units	Normality (Shapiro Wilk test)	Statistical test	p value	F/t/z/R ETC value	
**5f**	sAPPβ relative levels (aged mice, soluble fractions)	Vehicle	5	Optical density (sAPPβ normalized with β-actin)	–	1	0,04367	A.U.	yes	Unpaired t test	0,0002	t=6,352, df=8	***
		LY2811376	5			0,3668	0,0896		yes				
**5g**	sAPPα relative levels (aged mice, soluble fractions)	Vehicle	5	Optical density (sAPPα normalized with β-actin)	–	1	0,08265	A.U.	yes	Unpaired t test	0,0131	t=3,176, df=8	*
		LY2811376	5			1,424	0,1047		yes				
**5h**	APP relative levels (aged mice, membrane/cytosolic fractions)	Vehicle	5	Optical density (APP normalized with β-actin)	–	1	0,06175	A.U.	yes	Unpaired t test	0,0933	t=1,905, df=8	
		LY2811376	5			0,8246	0,0683		yes				
**5k**	Contribution of GluN2B to NMDAR EPSCs (%)	Vehicle	6	Amplitude (5 minutes before and 25-20 min after ifenprodil perfusion)	Ifenprodil (5µM)	37,95	8,445	pA (%)	yes	Unpaired t test	0,0219	t=2,496, df=19	*
		LY2811376	15			20,07	3,118		yes				
**5o**	Contribution of GluN2B to NMDAR EPSCs (%)	PTB1-p	10	Amplitude (5 minutes before and 25-20 min after ifenprodil perfusion)	Ifenprodil (5µM)	39,72	5,128	pA (%)	yes	Unpaired t test	0,0008	t=4,002, df=18	***
		PTB2-p	10			13,49	4,079		yes				

Supplementary Figure 1 – GluN2B synaptic current component and relative levels in mouse hippocampus and human brain

Figure	Experiment	Condition	number of mice/ human subjects	number of cells	Measurement	Mean	SEM	Units	Normality (Shapiro Wilk test)	Statistical test	p value	F/t/z/R ETC value	pos hoc Test	Comparison	p value	
**S1a**	%Amplitude slow	Infant	_	44	Amplitude slow / Amplitude total	60,83	1,74	pA (%)	yes	Ordinary One-way ANOVA	<0.0001	F (2, 102) = 34,58	Uncorrected Fisher's LSD	Adult vs. Infant	<0.0001	****
		Adult		26		31,17	2,675		yes					Adult vs. Aged	0.0050	**
		Aged		35		42,37	3,272		yes					Infant vs. Aged	<0,0001	
**S1b**	GluN2A relative levels (mice, PSD fractions)	Infant	5	_	Optical density (GluN2A, no normalization)	0,4942	0,1928	A.U.	yes	Ordinary One-way ANOVA	0,0444	F (2, 12) = 4,084	Uncorrected Fisher's LSD	Adult vs. Infant	0,0899	
		Adult	5			1	0,1928		yes					Adult vs. Aged	0,3521	
		Aged	5			1,265	0,1961		yes					Infant vs. Aged	0,0157	*
**S1c**	GluN2B relative levels (mice, PSD fractions)	Infant	5	_	Optical density (GluN2B, no normalization)	1,54	0,3136	A.U.	yes	Ordinary One-way ANOVA	0,2822	F (2, 12) = 1,408	_	_	_	_
		Adult	5			1	0,2148		yes							
		Aged	5			1,267	0,1052		yes							
**S1e**	p-GluN2B/GluN2B relative ratio (mice, PSD fractions)	Infant	5	_	Optical density (p-GluN2B/GluN2B)	0,312	0,08779	A.U.	yes	Ordinary One-way ANOVA	<0,0001	F (2, 14) = 41,04	Uncorrected Fisher's LSD	Adult vs. Infant	<0,0001	****
		Adult	6			1	0,04667		yes					Adult vs. Aged	0,5057	
		Aged	6			1,059	0,05542		yes					Infant vs. Aged	<0,0001	****
**S1g**	GluN2A relative levels (mice, total lysates)	Infant	8	_	Optical density (GluN2A normalized with b-actin)	0,112	0,02828	A.U.	yes	Kruskal-Wallis test	0,0002	Kruskal-Wallis statistic = 16,81	Uncorrected Dunn's test	Adult vs. Infant	<0,0001	****
		Adult	8			1	0,0826		yes					Adult vs. Aged	0,2293	
		Aged	8			0,826	0,147		no					Infant vs. Aged	0,0052	**
**S1h**	GluN2B relative levels (mice, total lysates)	Infant	8	_	Optical density (GluN2B normalized with b-actin)	0,6719	0,08911	A.U.	yes	Ordinary One-way ANOVA	0,019	F (2, 21) = 4,813	Uncorrected Fisher's LSD	Adult vs. Infant	0,0067	**
		Adult	8			1	0,06617		yes					Adult vs. Aged	0,0417	*
		Aged	8			0,7633	0,0745		yes					Infant vs. Aged	0,412	
**S1i**	GluN2B/GluN2A relative ratio (mice, total lysates)	Infant	8	_	Optical density (GluN2B/GluN2A)	8,071	1,66	A.U.	yes	Ordinary One-way ANOVA	<0,0001	F (2, 21) = 18,00	Uncorrected Fisher's LSD	Adult vs. Infant	<0,0001	****
		Adult	8			1	0,04618		yes					Adult vs. Aged	0,9883	
		Aged	8			1,02	0,1158		yes					Infant vs. Aged	<0,0001	****
**S1k**	GluN2A relative levels (mice, IP PSD-95)	Infant	4	_	Optical density (normalized with IP PSD95)	0,1714	0,02765	A.U.	yes	Ordinary One-way ANOVA	<0,0001	F (2, 9) = 85,28	Uncorrected Fisher's LSD	Adult vs. Infant	<0,0001	****
		Adult	4			1	0,08406		yes					Adult vs. Aged	0,1912	
		Aged	4			1,111	0,03802		yes					Infant vs. Aged	<0,0001	****
**S1l**	GluN2B relative levels (mice, IP PSD-95)	Infant	4	_	Optical density (normalized with IP PSD95)	1,163	0,2001	A.U.	yes	Ordinary One-way ANOVA	0,6946	F (2, 9) = 0,3796	_	_	_	_
		Adult	4			1	0,08777		yes							
		Aged	4			1,098	0,07269		yes							
**S1m**	GluN2B/GluN2A relative ratio (mice, IP PSD-95)	Infant	4	_	Optical density (GluN2B/GluN2A)	6,935	0,943	A.U.	yes	Ordinary One-way ANOVA	<0,0001	F (2, 9) = 39,56	Uncorrected Fisher's LSD	Adult vs. Infant	<0,0001	****
		Adult	4			1	0,03512		yes					Adult vs. Aged	0,9851	
		Aged	4			0,9852	0,04674		yes					Infant vs. Aged	<0,0001	****
**S1o**	Correlation between GluN2B relative levels and age (humans)	_	12	_	Optical density (GluN2B normalized with GAPDH)	_	_	A.U.	_	Pearson Correlation	0,4872	R^2=0,04945	_	_	_	_

Supplementary Figure 2 – Synaptic APP expression and co-immunoprecipitation with NMDARs. Electrophysiological controls of APP-NMDA interaction.

Figure	Experiment	Condition	number of animals	number of cells	Measurement	Treatment	Mean	SEM	Units	Normality (Shapiro Wilk test)	Statistical test	p value	F/t/z/R ETC value	pos hoc Test	Comparison	p value	
**S2a**	APP relative levels (mice, PSD fractions)	Infant	8	_	Optical density (APP, no normalization)	_	2,106	0,3069	A.U.	yes	Ordinary One-way ANOVA	0,0005	F (2, 21) = 11,19	Uncorrected Fisher's LSD	Adult vs. Infant	0,0006	***
		Adult	8				1	0,09333		yes					Adult vs. Aged	0,8865	
		Aged	8				0,9602	0,1027		yes					Infant vs. Aged	0,0004	***
**S2f**	GluN2B/GluN2A relative ratio (mice, IP APP)	Infant	4	_	Optical density (GluN2B/GluN2A)	_	5,126	1,988	A.U.	yes	Ordinary One-way ANOVA	0,049	F (2, 9) = 4,296	Uncorrected Fisher's LSD	Adult vs. Infant	0,0319	*
		Adult	4				1	0,0972		yes					Adult vs. Aged	0,9968	
		Aged	4				0,9934	0,1007		no					Infant vs. Aged	0,0317	*
**S2g**	Reduction in NMDAR EPSCs after APP blocking (%)	Ab 2.5nM	_	14	Amplitude (55-60 min comparing to baseline (15-20min)	APPCt, clone Y188 (0-60min) / control	21,11	4,47	pA (%)	yes	Unpaired t test	0,1197	t=1,629, df=19	_	_	_	_
		Ab 5nM		7			33,93	6,645		yes							

Supplementary Figure 3 – APP effects on PSD-95, GluN2B cluster size and GluN2B/PSD-95 total levels

Figure	Experiment	Condition	Number of dendrites	Number of independent neuronal cultures	Measurement	Mean	SEM	Units	Normality (Shapiro Wilk test)	Statistical test	p value	F/t/z/R ETC value	
**S3a**	Relative average size of GluN2B particles (%)	shCTR	39	3	Average size of GluN2B particles	100	16,54	μm^2 (%)	no	Mann Whitney test	0,1432	Mann-Whitney U = 613,5	
		shAPP	39	3		133,4	19,71		no				
**S3b**	Relative GluN2B fluorescence density (%)	shCTR	39	3	Mean GluN2B fluorescence intensity x GluN2B area	100	17,42	A.U.	no	Mann Whitney test	0,2241	Mann-Whitney U = 638	
		shAPP	39	3		127,8	18,39		no				
**S3c**	Relative average size of PSD-95 particles (%)	shCTR	39	3	Average size of PSD-95 particles	100	2,997	μm^2 (%)	yes	Unpaired t test	0,0026	t=3,114, df=76	**
		shAPP	39	3		87,39	2,72		yes				
**S3d**	Relative PSD-95 fluorescence density (%)	shCTR	39	3	Mean PSD-95 fluorescence intensity x PSD-95 area	100	7,541	A.U.	yes	Mann Whitney test	0,0703	Mann-Whitney U = 579	
		shAPP	39	3		80,96	7,817		no				

Supplementary Figure 4 – APP processing throughout aging in mouse and human brain.

Figure	Experiment	Condition	number of mice/ human subjects	Measurement	Mean	SEM	Units	Normality (Shapiro Wilk test)	Statistical test	p value	F/t/z/R ETC value	pos hoc Test	Comparison	p value	
		Infant	8		0,8007	0,126		yes					Adult vs. Infant	0,1588	
**S4a**	CTFs relative levels (mice, whole lysates)	Adult	10	Optical density (CTFs normalized with β-actin)	1	0,03324	A.U.	yes	Ordinary One-way ANOVA	0,0002	F (2, 28) = 12,13	Uncorrected Fisher's LSD	Adult vs. Aged	0,0023	**
		Aged	13		1,409	0,09374		yes					Infant vs. Aged	<0,0001	****
		Infant	8		0,6851	0,1232		yes					Adult vs. Infant	0,0556	
**S4b**	AICD relative levels (mice, whole lysates)	Adult	8	Optical density (AICD normalized with β-actin)	1,036	0,04517	A.U.	yes	Ordinary One-way ANOVA	0,0155	F (2, 25) = 4,946	Uncorrected Fisher's LSD	Adult vs. Aged	0,3635	
		Aged	12		1,184	0,126		yes					Infant vs. Aged	0,0045	**
		Infant	6		0,5975	0,1332		yes					Adult vs. Infant	0,0733	
**S4c**	CTF β/α relative ratio (mice, whole lysates)	Adult	8	Optical density (CTF β/α)	1	0,07437	A.U.	yes	Ordinary One-way ANOVA	0,0122	F (2, 22) = 5,417	Uncorrected Fisher's LSD	Adult vs. Aged	0,174	
		Aged	11		1,259	0,1541		yes					Infant vs. Aged	0,0034	**
		Infant	6		1,097	0,1172		yes							
**S4d**	C99 relative levels (mice, whole lysates)	Adult	6	Optical density (C99 normalized with β-actin)	1	0,1022	A.U.	yes	Ordinary One-way ANOVA	0,7254	F (2, 15) = 0,3281	_	_	_	_
		Aged	6		1,1	0,07348		yes							
**S4e**	Correlation between CTFs relative levels and age (humans, whole lysates)	-	20	Optical density (CTFs normalized with GAPDH)	-	-	A.U.	-	Pearson Correlation	0,4633	R^2=0,03026	_	_	_	_
**S4f**	Correlation between AICD relative levels and age (humans, whole lysates)	-	20	Optical density (AICD normalized with GAPDH)	-	-	A.U.	-	Pearson Correlation	0,008	R^2=0,3303	_	_	_	_
**S4g**	Correlation between CTF β/α relative ratio and age (humans, whole lysates)	-	20	Optical density (CTF β/α)	_	_	A.U.	_	Pearson Correlation	0,1613	R^2=0,106	_	_	_	_
		Adult	3		1,574	0,03345		yes					Adult vs. Aged	0,0005	***
**S4i**	APP relative levels (human)	Aged	4	Optical density (APP normalized with GAPDH)	1	0,06631	A.U.	yes	Ordinary One-way ANOVA	0,0003	F (2, 8) = 25,33	Uncorrected Fisher's LSD	Adult vs. AD	0,0001	***
		AD	4		0,8732	0,0844		yes					Aged vs. AD	0,2194	
		Adult	3		0,7801	0,151		yes							
**S4j**	C-terminal fragments/APP relative levels (human)	Aged	4	Optical density (CTFs/APP)	1	0,2097	A.U.	yes	Kruskal-Wallis test	0,6038	Kruskal-Wallis statistic=1,136	_	_	_	_
		AD	4		1,038	0,4906		no							
		Adult	3		0,3834	0,06053		yes							
**S4k**	AICD/APP relative levels (human)	Aged	4	Optical density (AICD/APP)	1	0,2928	A.U.	yes	Ordinary One-way ANOVA	0,1489	F (2, 8) = 2,439	_	_	_	_
		AD	4		1,412	0,4005		no							

NZYColour Protein Marker I (NZYTech)

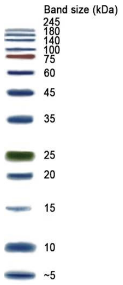

Fig 1g - PSD fractionation, mice

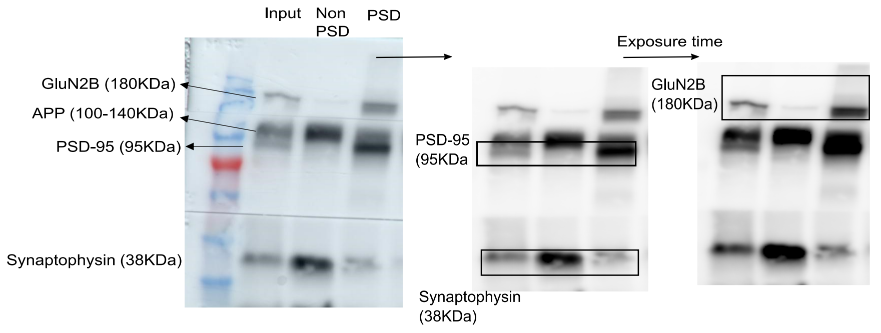

Fig 1h - NMDARs in PSD fractions, mice

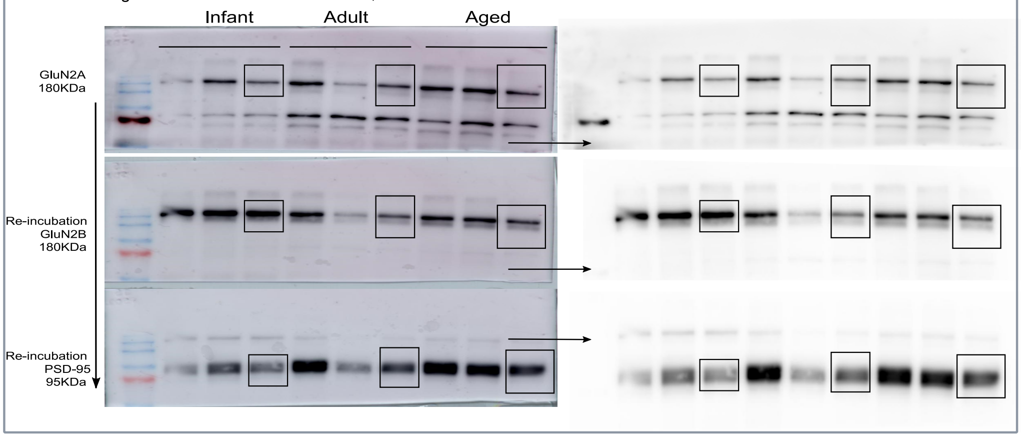

Fig 2a - APP in PSD fractions, mice

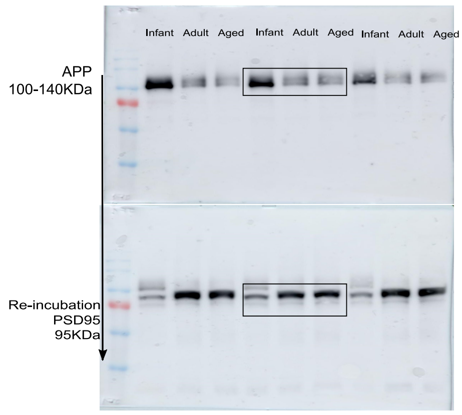

Fig 2c - CoIP APP in synaptosomes, mice

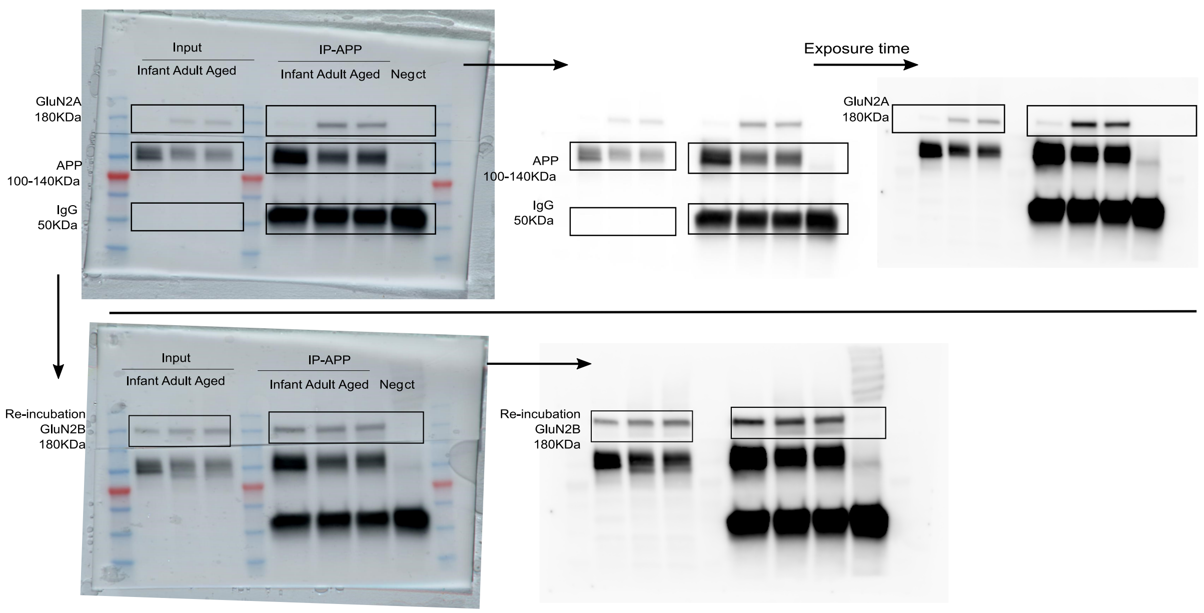

NZYColour Protein Marker I
(NZYTech)

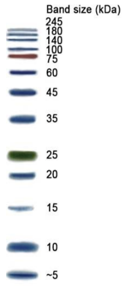

Fig 2d - CoIP APP in total lysates, human

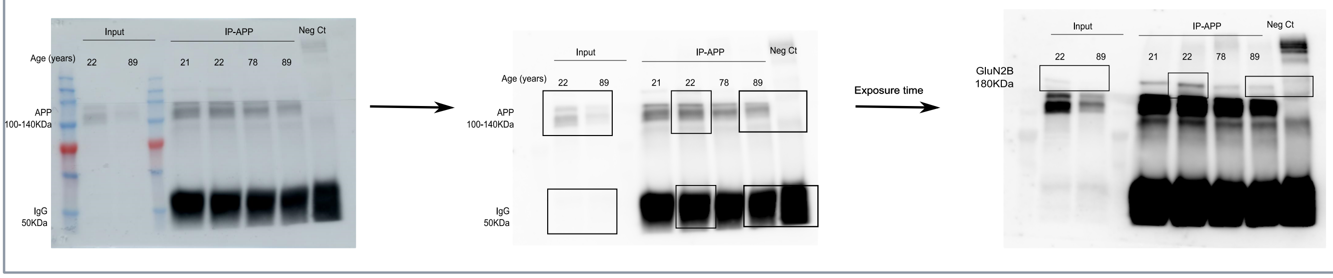

Fig 4a - APP in whole lysates, mice

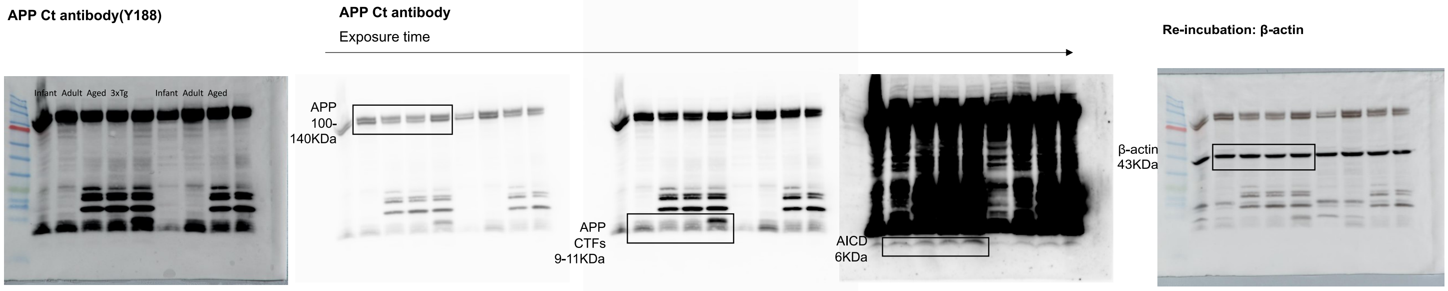

Fig 4e- APP C99 levels in whole lysates, mice

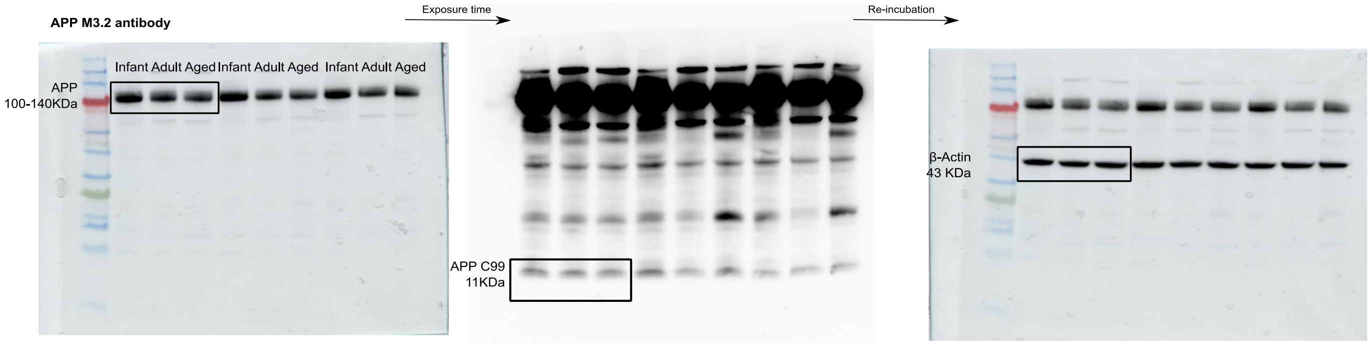

Fig 4g - APP in whole lysates, human

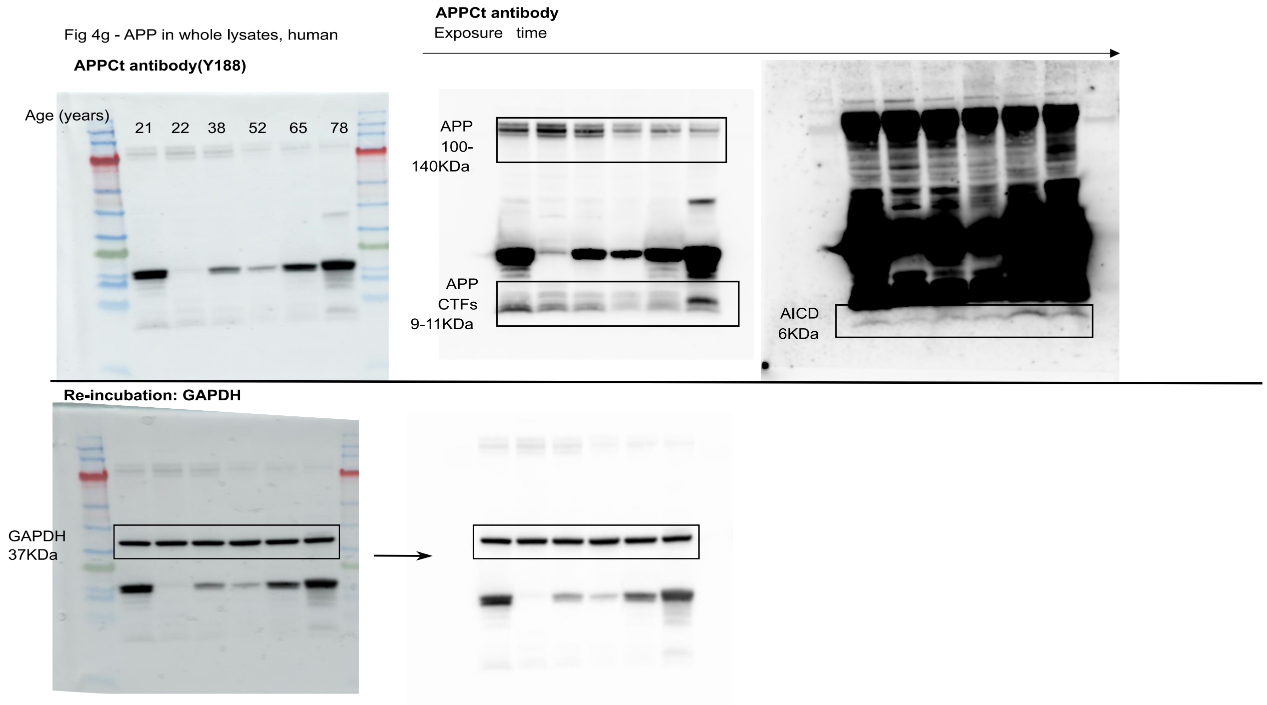

Figure 5c - sAPPβ levels in aged mice treated with LY2811376

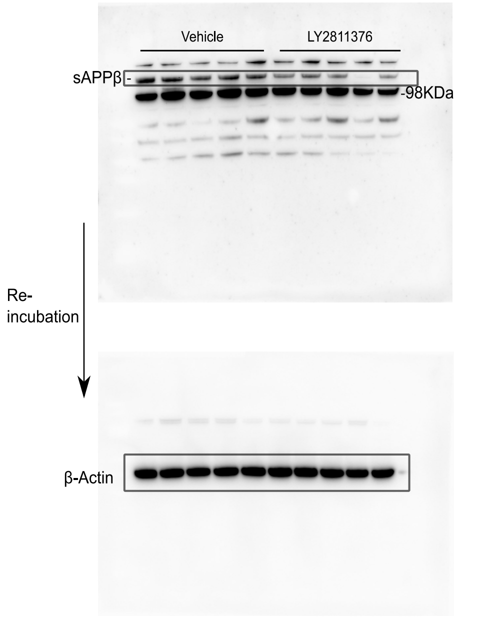

Figure 5d - sAPPα levels in aged mice treated with LY2811376

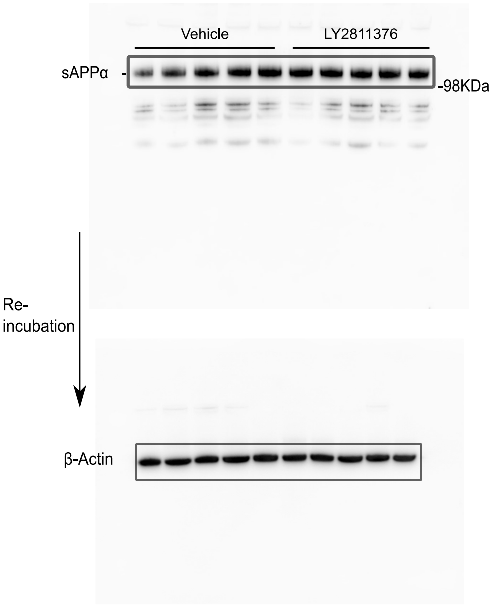

Figure 5e - APP levels in aged mice treated with LY2811376

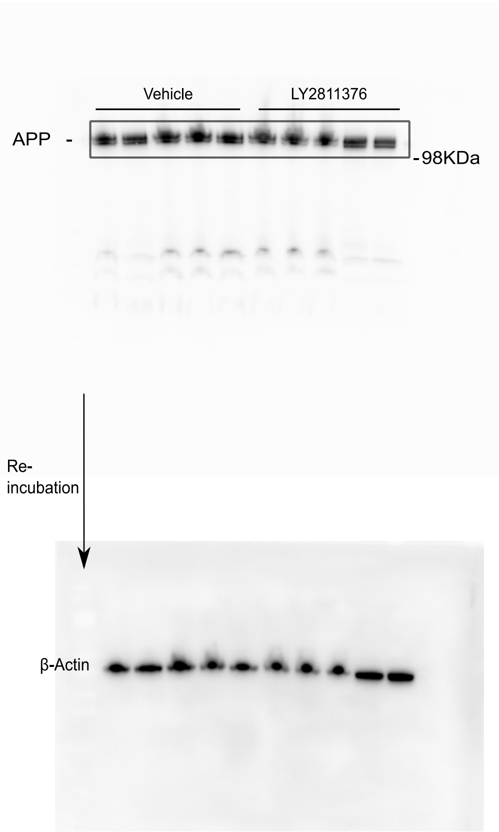

NZYColour Protein Marker I (NZYTech)

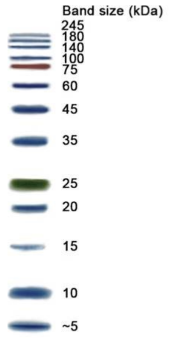

Supplementary Fig 1d - pGluN2B/ GluN2B ratio in PSD fractions, mice

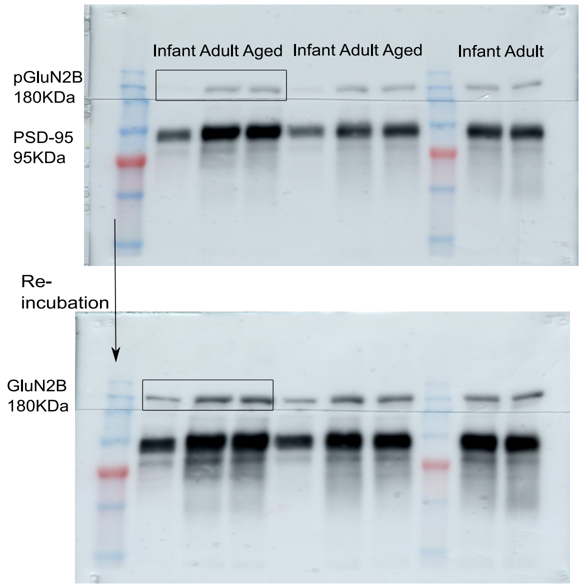

Supplementary Fig 1f - NMDAR levels in whole lysates, mice

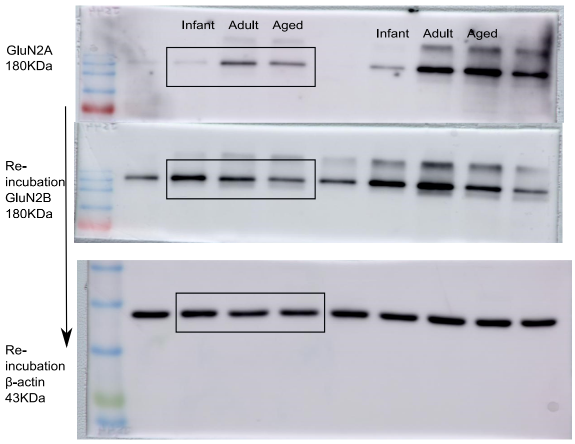

Supplementary Fig 1j - CoIP PSD95 in whole lysates, mice

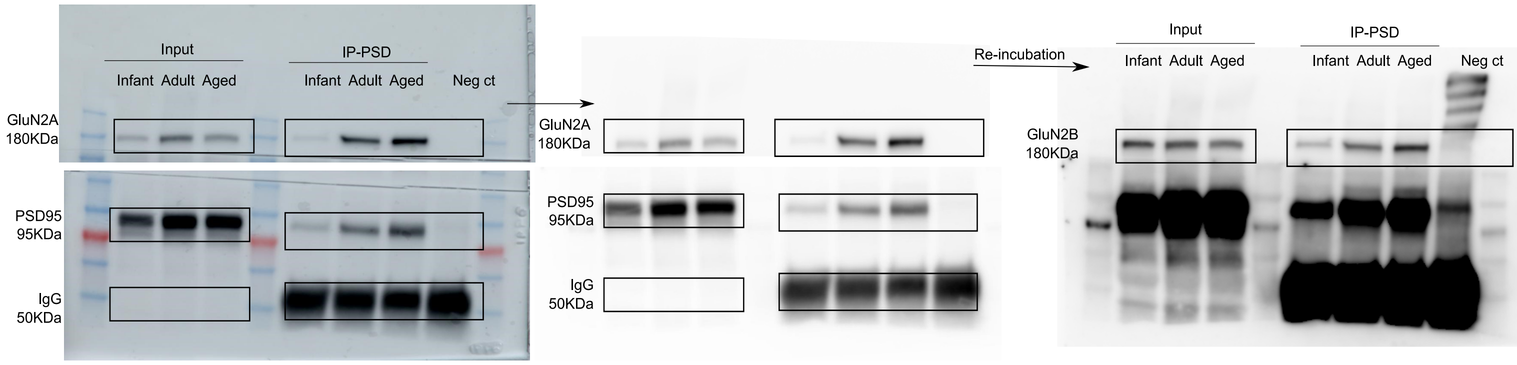

Supplementary Fig 1n- GluN2B in whole lysates, humans

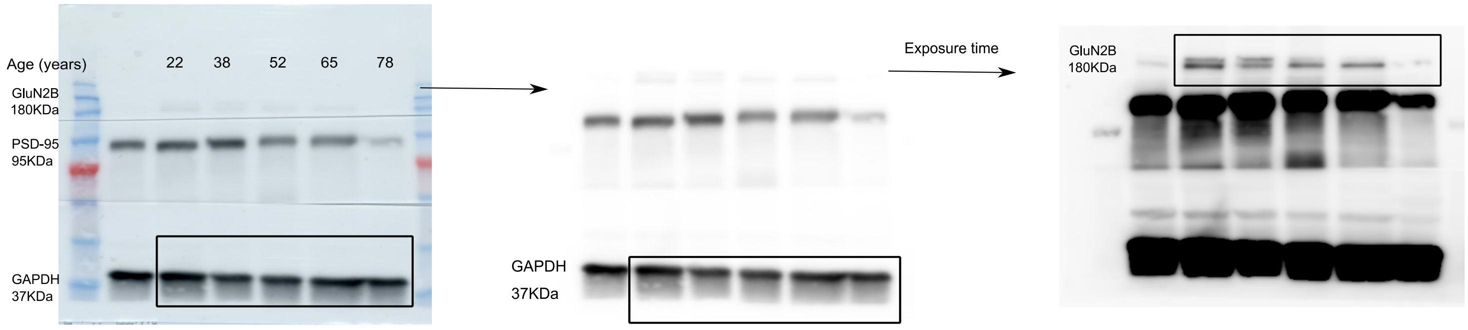

NZYColour Protein
Marker I
(NZYTech)

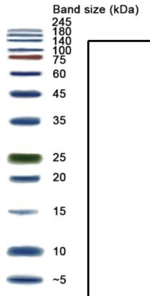

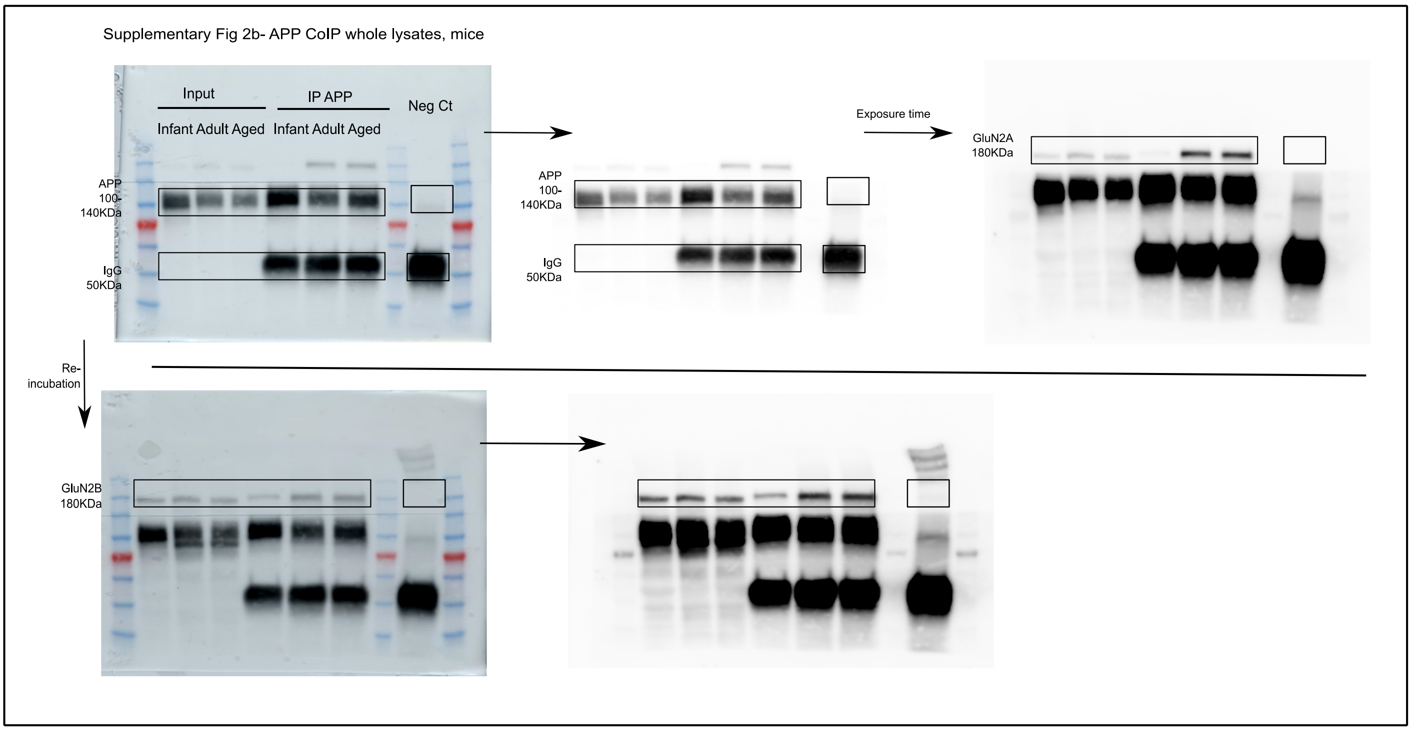

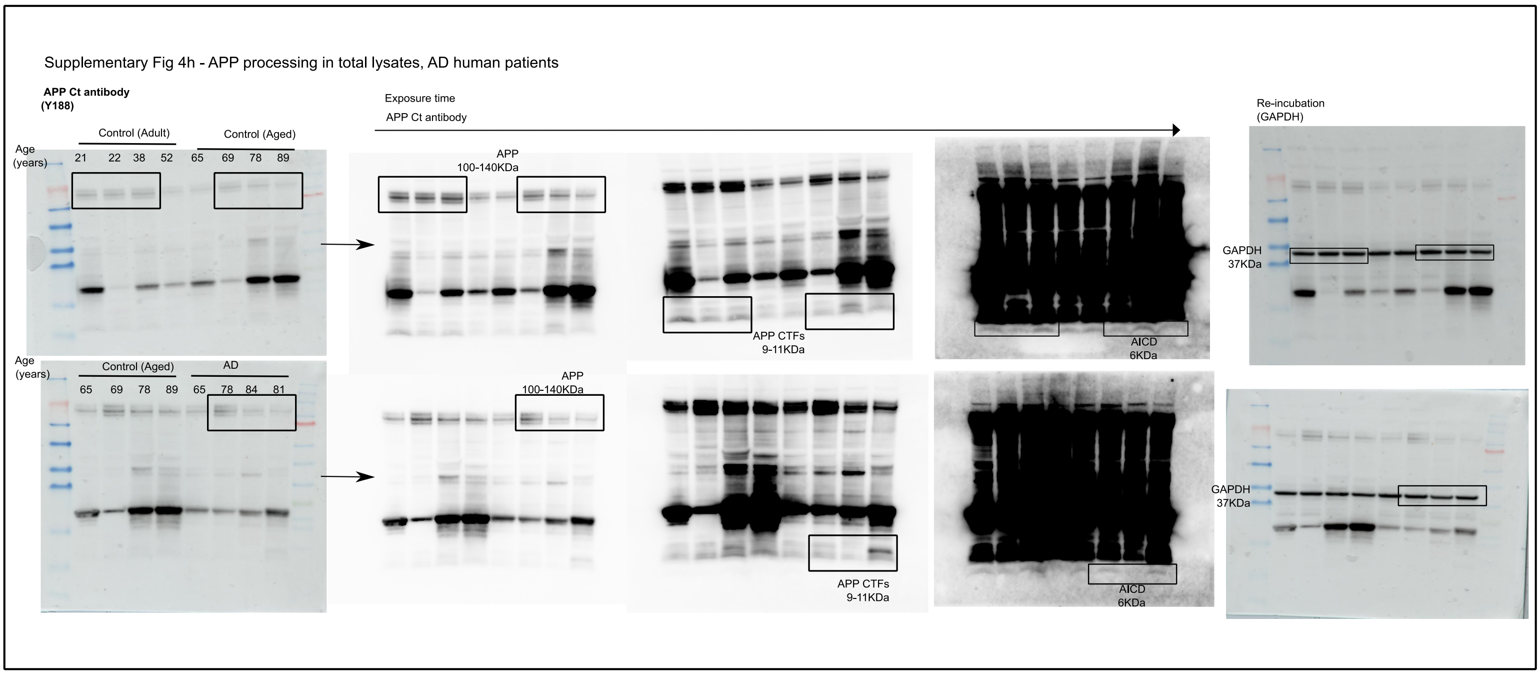

Supplement: Supplementary file 1 — Appendix S1. [file ACEL-22-e13778-s001.pdf]
